# SOX15 transcriptionally increases the function of *AOC1* to modulate ferroptosis and progression in prostate cancer

**DOI:** 10.1038/s41419-022-05108-w

**Published:** 2022-08-03

**Authors:** Yinghui Ding, Yuankang Feng, Zhenlin Huang, Yu Zhang, Xiang Li, Ruoyang Liu, Hao Li, Tao Wang, Yafei Ding, Zhankui Jia, Jinjian Yang

**Affiliations:** Department of Urology, the First Affiliated Hospital of Zhengzhou University, 450052 Zhengzhou, China

**Keywords:** Prostate cancer, Transcriptional regulatory elements

## Abstract

Amine oxidase copper-containing 1 (*AOC1*) is considered an oncogene in many types of tumors. Nevertheless, there have been no investigations of *AOC1* and its regulatory mechanism in prostate cancer. Here, we reveal a novel action of *AOC1* and a tumor suppressor mechanism in prostate cancer. *AOC1* is downregulated in prostate cancer. Abatement of *AOC1* in prostate cancer tissue is positively correlated with the tumor size, lymph node metastasis, and Gleason score for prostate cancer. Conversely, high expression of *AOC1* is significantly associated with reduced proliferation and migration in prostate cancer both in vitro and in vivo. We show that the anticancer effect of *AOC1* is mediated by its action on spermidine which leads to the activation of reactive oxygen species and ferroptosis. *AOC1* expression in prostate cancer is positively regulated by the transcription factor *SOX15*. Therefore, *SOX15* can transcriptionally promote *AOC1* expression and strengthen this effect. Targeting *AOC1* and *SOX15* may be promising for the treatment of prostate cancer.

## Introduction

Amine oxidase copper-containing 1 (*AOC1*) encodes amiloride-binding protein 1, which can bind amiloride or amiloride analogs [1]. Studies have shown that the preferred substrate of amiloride-binding protein 1 is diamines, particularly histamine [2]. Because of catalyzed oxidation of diamines to aldehydes, *AOC1* regulates many biological processes, such as tumor growth and development, inflammation, and neoplasm [3]. Karin et al. linked *AOC1* to kidney development [4]. Furthermore, *AOC1* has been demonstrated to promote gastric and colorectal cancer [5, 6], although, little is known about its role in other cancers.

*AOC1* functions using as substrate spermidine that is incipiently found in semen and later discovered in many natural plants [7]. In general, spermidine has a significant role in eukaryotic cell proliferation [8], inhibition of stem cell senescence, and decreasing blood pressure [9, 10]. Interestingly, Fan et al. indicated that although increasing spermidine uptake can inhibit oncogenesis, it can accelerate the progression of already-generated tumors [7].

In previous studies, transcription factor WT1 was shown to regulate *AOC1* expression; however, its association with *AOC1* in prostate cancer is not strong. To explore the expression regulation mechanism of *AOC1*, we expected to find a new transcription factor that can regulate *AOC1* expression. SOX15, as a transcription factor, has been widely demonstrated to inhibit tumor growth through the Wnt/β-catenin pathway [11–13] and to function with miR-96 to regulate androgen receptor signals during prostate cancer progression [14]. However, its mechanism of action in prostate cancer has not been rigorously investigated.

In the present study, we demonstrated that SOX15 positively regulates AOC1 expression and that *AOC1* has a tumor suppressor function in prostate cancer by downregulating the proliferation and migration of prostate cancer cells via the decomposition of spermidine. *AOC1* downstream products such as H_2_O_2_—reactive oxygen species (ROS) are involved in ferroptosis. Ferroptosis is a non-apoptotic form of cell death found in many cancers [15, 16] triggered by ROS and lipid peroxidation [17]. Thus, the SOX15/AOC1/ROS axis might be a promising therapeutic target for prostate cancer.

## Materials and methods

### Data mining

We speculated on the differential expression of *AOC1* and its relationship with SOX15 using seven Gene Expression Omnibus (GEO) datasets (GSE46602, GSE71016, GSE28204, GSE27616, GSE38241, GSE114740, and GSE6752; https://www.ncbi.nlm.nih.gov/geo). Based on these GEO datasets, we analyzed differentially expressed genes of prostate cancer and normal prostate tissue using the online program GE02R based on the limma R package (http://www.ncbi.nlm.nih.gov/geo/geo2r/). False-positive data were amended by adjusting the *p*-value. Hence, we chose adjusted *p* < 0.05 and log FC > 1 or log FC < −1 as the significant data. Venn diagrams and volcano plots were used to distinguish the intersection of differentially expressed genes across these GEO datasets. To optimize the relationship between the expression of *AOC1* and *SOX15* and the prognostic value of prostate cancer, we supplemented the data with the Cancer Genome Atlas (TCGA) cohort, which was analyzed using the Gene Expression Profiling Interactive Analysis (GEPIA) database (http://gepia.cancer-pku.cn/). We obtained the protein–protein interaction networks of *AOC1*-encoded proteins (amiloride-binding protein 1) using the Search Tool for Recurring Instances of Neighboring Genes (STRING) (https://string-db.org/). Furthermore, we predicted the transcription factor of the *AOC1* promoter from the JASPAR CORE database (http://jaspar.genereg.net/).

### Tissue specimens

Ninety-four tissue samples were collected from prostate cancer patients between 2011 and 2013, at the First Affiliated Hospital of Zhengzhou University (Zhengzhou, China). The patients in this study received a definite diagnosis by pathology examination and had not undergone medical treatment. Written informed consent was obtained from every patient included in the study. The research was approved by the Research Ethics Committee of the First Affiliated Hospital of Zhengzhou University (ZZU-LAC20210924 (15)).

### Cell culture and transfection

Human prostate cancer cell lines DU145 and 22Rv1 were purchased from the American Type Culture Collection (LGC Standards, London, England). Both cell lines were periodically certified and tested for mycoplasma contamination. The cells were cultured in Roswell Park Memorial Institute (RPMI) 1640 medium (Thermo Fisher, Waltham, MA, USA) containing 10% fetal bovine serum (FBS; Invitrogen, Carlsbad, CA, USA) and 1% penicillin–streptomycin liquid (Gibco, Carlsbad, CA, USA) at 37 °C with 5% CO_2_. Cells were cultured to 60–80% confluence; then, they were transfected using jetPRIME^®^ in vitro DNA and siRNA transfection reagent (Polyplus-transfection, Strasbourg, France) according to the manufacturer’s instructions.

### Quantitative real-time polymerase chain reaction

Total RNA was extracted using the TRIzol^®^ reagent (Invitrogen). The cDNA was prepared with NovoScript^®^ Plus All-in-one 1st Strand cDNA Synthesis SuperMix (gDNA Purge). (Novoprotein, Shanghai, China). Gene transcripts were quantitated using the QuantStudio Three Real-Time PCR System (Thermo Fisher) using the NovoStart^®^ SYBR qPCR SuperMix Plus (Novoprotein), with GAPDH as an internal control. The primers sequence used during our quantitative real-time polymerase chain reaction (qRT-PCR) test is shown in Supplementary Table 1.

### Immunohistochemistry

Paraffin-embedded tissues were sliced into 5-μm sections and observed for AOC1 expression after immunohistochemistry staining. These sections were dyed with diluted anti-AOC1 antibody (1:500; ab231558; Abcam, Cambridge, UK) and incubated at 4 °C overnight; then, they were washed three times with fresh phosphate-buffered saline (PBS) solutions and immediately incubated with biotin-labeled second antibody for immunohistochemistry (GB23303; Servicebio, Wuhan, China) for 50 min at room temperature. The positive cells were monitored using diaminobenzidine solution (K5007; DAKO, Santa Clara, CA, USA) by direct viewing. The processed slides were observed using a light microscope (Leica DM2700). Every immunohistochemistry result was read blindly and independently by two senior pathologists.

The positive cell scores were defined as follows: 1 point, 1–9% positive cells; 2 points, 10–50% positive cells; 3 points, 51–80% positive cells; and 4 points, >80% positive cells. We defined the staining intensity scores as follows: 1 point, negative staining; 2 points, weak staining; 3 points, moderate staining; and 4 points, strong staining. Our final immunoreactivity score was the product of the positive cell score and staining intensity score.

### Western blot

The total protein content in tissues and cells was extracted using RIPA buffer (Solarbio, Beijing, China) supplemented with 1% PMSF (Solarbio). The quantification was completed with the help of the BCA Protein Assay Kit (Solarbio). We used SDS–PAGE gels prepared from the PAGE Gel Fast Preparation Kit (Epizyme, shanghai, China) to separate the total protein (20 μg). Isolates were immediately anchored onto nitrocellulose membranes (Millipore, Danvers, MA, USA). A blocking solution for 1 h at room temperature was necessary for more specific antibody binding. The blotted nitrocellulose membranes were incubated with the primary antibody, anti-AOC1 antibody (1:1000; ab231558; Abcam, UK), anti-TFR antibody (1:1000; ab214039; Abcam), anti-TF antibody (1:1000; ab277635; Abcam,), anti-FTH1 antibody (1:1000; ab75973, Abcam), and anti-SOX15 antibody (1:100; sc-166964; Santa Cruz, Dallas, TX) overnight at 4 °C. After washing with Tris-buffered saline with Tween, the nitrocellulose membranes were incubated with goat anti-rabbit IgG (IRDye^®^ 800CW; 1:5000; ab216773; Abcam) or goat anti-mouse IgG (IRDye^®^ 800CW). Anti-GAPDH antibody (1:1000; ab9485; Abcam) was used as a loading control. The Odyssey CLx Infrared Imaging System (Gene Company Limited, Hong Kong, China) was used to detect the target protein bands.

### Cell counting kit-8 assay

The cell counting kit-8 (CCK8) assay (Dojindo, Tokyo, Japan) was used to assess the proliferative viability of tumor cell lines. Three thousand cells were cultured in 96-well plates filled with 100 μL RPMI-1640 and 10% FBS for 96 h. Then, 10 µL of the CCK8 reagent was supplemented to each well 4 h after cell placement. The absorbance in each well was measured with a DNM-9606 microplate reader (Perlong, Beijing, China) at 450 nm every 24 h.

### EdU assay

We used the EdU assay kit (RiboBio, Guangzhou, China) according to the manufacturer’s protocol to verify the proliferation abilities of the cells. The treated cells were placed in 96‐well plates at 4 × 10^4^ cells/well overnight. Then, cells were treated with 50 μmol/L EdU and incubated for 6 h at 37 °C, followed by fixation with paraformaldehyde solution for 15 min and permeabilization with 0.5% Triton X‐100 for 20 min. Incubation with 100 μL of 1 × Apollo^®^ reaction cocktail performed for 30 min at room temperature and protection from light was the most important conditions. Finally, the nuclei of the cells were dyed with DIPA for 5 min and observed by fluorescence microscopy.

### Colony formation assay

Approximately 1200 22Rv1 and DU145 cells were placed on the plate. The RPMI-1640 with 10% FBS was replaced every 5 days with a fresh medium. After culturing for 15 days, the cells were fixed with 1 mL of paraformaldehyde for 30 min; then, they were washed three times with PBS. Dyeing with 0.1% crystal violet for 30 min was the most important step. The clones were conscientiously counted after being washed and dried at room temperature.

### Wound-healing assay

A wound-healing assay was used to confirm the migration ability of cell lines. Altogether, 1 × 10^6^ prostate cancer cells were placed and cultured in RPMI-1640 with FBS. When the cell density was 80–100%, a wound was made in the cell monolayer, and the prime image was immediately photographed. Then, cells placed in six-well plates were cultured for another 12 or 48 h, and the corresponding images were photographed immediately. The area of cell migration was our primary outcome measure used to analyze the migration ability of cell lines.

### Transwell assay

The cell migration ability was also assessed using the Transwell assay with 8-μm pores (Corning Inc., Corning, NY, USA) as a supplement. The cells were collected by trypsin digestion and placed into the upper chambers with 1 × 10^5^ cells; then, the medium in each well was replenished to 200 μL. The upper chambers were placed in a 24-well plate after each well of RPMI-1640 medium with 20% FBS. After being incubated for 24 h at 37 °C and 5% CO_2_, the upper chambers were stained, observed, and analyzed.

### H_2_O_2_ assay

The content of H_2_O_2_ in the cells was analyzed using a content detection kit (Solarbio, Beijing, China). The cells were collected in a centrifuge tube, and the supernatant was discarded after centrifugation; 1 mL reagent I was added per 5 × 10^6^ cells, and cells were broken using ultrasonic waves (power, 20%; ultrasonic waves, 3 s; interval, 10 s; repetitions, 30) followed by centrifugation at 8000×*g* for 10 min at 4 °C. Finally, the supernatant was placed on ice before testing. The spectrophotometer or microplate reader was preheated for more than 30 min and the wavelength was set to 415 nm; it was adjusted to zero using distilled water. Reagents II, III, and IV were placed in a water bath at 37 °C for more than 10 min. Samples and reagents were added in order according to the instruction for use and allowed to stand at room temperature for 5 min; then, 200 μL was transferred to a 96-well plate to determine the absorbance at 415 nm and calculate ∆*A*, from which the H_2_O_2_ content was calculated.

### ROS fluorescence probe assay

The H2DCFDA (DCFH-DA, DCFH) ROS fluorescence probe (MKBio, Shanghai, China) was used to complement and validate the content of intracellular ROS. A total of 2.5 × 10^5^ cells to be tested in each well were placed in a six-well plate, and the culture medium was extracted before adding the 10 mM of working solution with PBS. They were incubated at room temperature for 40 min. The staining working solution was sucked out and washed once with preheated cell culture medium. The pre-warmed cell culture medium was added again before evaluation using a microscope.

### MDA assay

A micro-malondialdehyde (MDA) assay kit (Solarbio) was purchased to detect the level of lipid oxidation in cell membranes. The cells were collected in a centrifuge tube, and the supernatant was discarded after centrifugation; 1 mL of reagent I was added per 5 million cells. Ultrasonic waves (power, 20%; ultrasonic waves, 3 s; interval, 10 s; repetitions, 30 times) were used to break the bacteria or cells before centrifugation at 8000 × *g* for 10 min at 4 °C; the supernatant was collected and placed on ice before testing. The spectrophotometer or microplate reader was preheated for more than 30 min and the wavelength was set to 415 nm; this was adjusted to zero with distilled water. The samples and reagents I, II, and III were added in order according to the IFU. After the mixture was kept warm in a water bath at 100 °C for 60 min (capped tightly to prevent water loss), it was cooled in an ice bath at room temperature and centrifuged at 10,000 × *g* for 10 min.

A 200-μL aliquot of the supernatant was pipetted into a 96-well plate to measure the absorbance at 450, 532, and 600 nm to determine the Δ*A*, from which the MDA content was calculated.

### Liperfluo fluorescence probe assay

The Liperfluo fluorescence probe (Dojindo, Beijing, China) was used to complement and validate the intracellular lipid peroxide content. A total of 2.5 × 10^5^ cells to be tested in each well were placed in a six-well plate, and the culture medium was extracted before adding the 10 mM of working solution with PBS and incubating at room temperature for 40 min. The staining working solution was sucked out and washed once with preheated cell culture medium. The cells were resuspended with pancreatin and later detected using a flow cytometer (excitation wavelength, 488 nm; emission wavelength, 515–545 nm).

### In vivo tumor xenograft model

According to the requirements of statistical methods, we randomly selected 5 mice in each group for the experiment, which is also consistent with most articles. The severe combined immunodeficiency mice (4–5 weeks old; male) used for in vivo transplantation experiments were purchased from Sibeifu Company (Beijing, China). The 22Rv1 cells (6 × 10^6^) infected with vector and shControl, *AOC1* and shControl, and *AOC1* and siSOX15 were dispersed in 50 μL PBS and 50 μL Matrigel (Corning) and inoculated subcutaneously into the dorsal flank of the mice. A power analysis was performed to calculate the sample size needed for our experiments. All animals were randomly assigned to different groups. Observation and measurement of the tumor size were performed every 3 days, and the tumor was removed and weighed on day 33.

Experimental procedures were performed in accordance with the guidelines established by the National Institutes of Health of China and approved by the Ethics Committee for Animal Experiments of the First Affiliated Hospital of Zhengzhou University, Zhengzhou, China.

### Statistical analysis

All data were analyzed using GraphPad Prism 7 Software (GraphPad, San Diego, CA, USA) and SPSS v. 24.0 software (2010; IBM, Chicago, IL, USA). Unless otherwise stated, two-tailed Student’s *t*-test or an analysis of variance was used to assess significance. Three replicates were performed for every experiment. *P* < 0.05 indicated statistical difference.

## Results

### The expression of *AOC1* was downregulated in prostate cancer

**Fig. 1:**
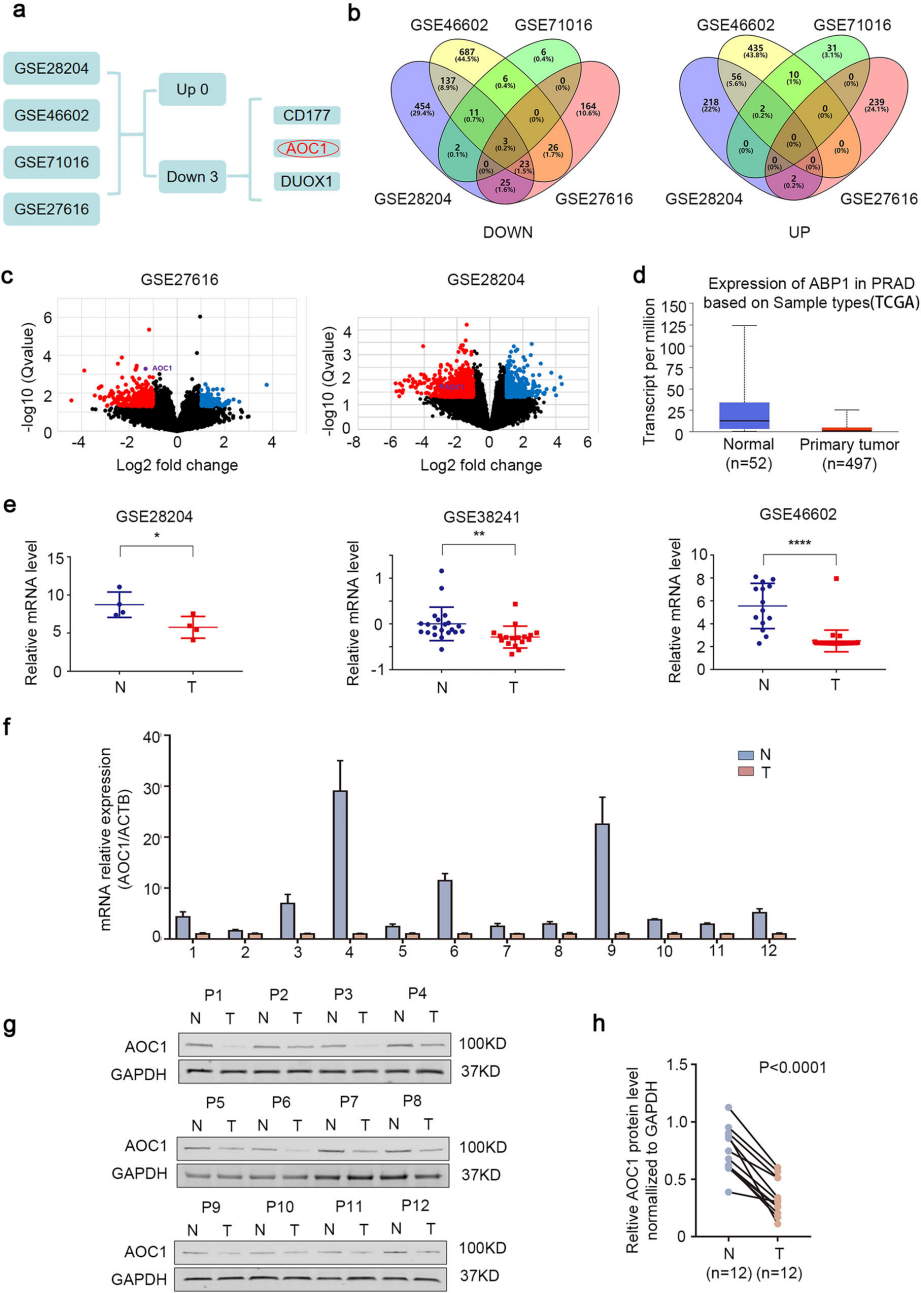
The expression of *AOC1* was considered to be downregulated in prostate cancer. **a** The process of selecting the *AOC1* gene. **b**, **c** Differentially expressed genes in prostate cancer tissues from four GEO datasets (GSE46602, GSE71016, GSE28204, and GSE27616), in the form of a Wayne diagram (**b**) and a volcano diagram (**c**), respectively. **d** *AOC1* expression in prostate cancer based on TCGA database. **e** *AOC1* expression in prostate cancer based on GEO datasets (GSE28204, GSE38241, and GSE46602). **f**–**h** RT-qPCR (**f**) and Western blot analysis (**g**, **h**) show the *AOC1* expression in prostate cancer and normal tissues. GAPDH served as an internal reference. *P* < 0.0001, paired *t*-test.

Although substantial progress has been made in the research of prostate cancer, metastasis, drug resistance, and transformation to castration-resistant prostate cancer (CRPC) remind us that further studies of prostate cancer are warranted. Therefore, we mined four reported GEO databases related to prostate cancer. Three genes (*CD177*, *AOC1*, and *DUOX1*) had significantly low expression in all four GEO datasets (GSE46602, GSE71016, GSE28204, and GSE27616) (Fig. 1a–c, Supplementary Fig. 1a, b). The roles of CD177 and DUOX1 in prostate cancer have been comprehensively reported; however, how *AOC1* is involved in prostate cancer remains unclear. Consistent with previous results, TCGA data also proved that *AOC1* expression is reduced in prostate cancer (Fig. 1d); this was also shown by the GEO dataset results (Fig. 1e, Supplementary Fig. 1c, d). We collected 94 surgical and puncture prostate cancer specimens in clinical practice and randomly selected 12 pairs of prostate cancer tissues and normal tissues for qRT-PCR. The results showed that the mRNA expression of *AOC1* in prostate cancer was lower than that in normal tissues (Fig. 1f). This was verified by the Western blot of AOC1 protein expression (Fig. 1g, h). These results adequately showed that *AOC1* expression is decreased in prostate cancer, suggesting that *AOC1* may have a role in inhibiting prostate tumor progression.

### Expression of *AOC1* is correlated with clinical outcomes of prostate cancer

**Fig. 2:**
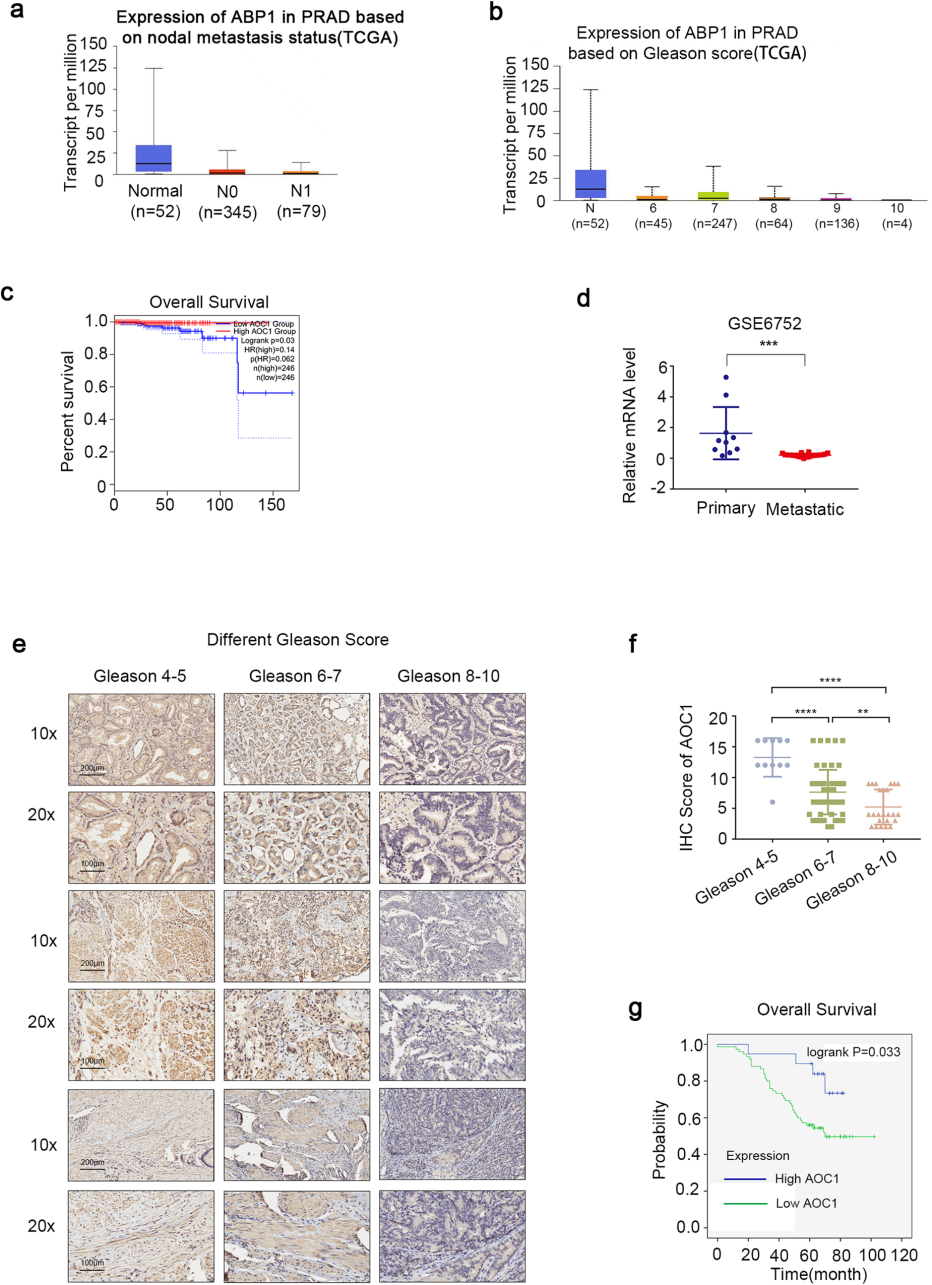
Expression of *AOC1* is correlated with clinical outcomes of prostate cancer. **a** TCGA database showed *AOC1* expression in prostate cancer and correlation with lymph nodes metastasis in. **b** TCGA database showed *AOC1* expression in prostate cancer and correlation with Gleason score. **c** TCGA data showed prostate cancer patients' *AOC1* expression correlation with prognosis. **d** GEO data (GSE6752) showed *AOC1* expression in primary and metastatic prostate cancer. **e**, **f** Images (**e**) and dot plots (**f**) of *AOC1* staining using tissue sections based on Gleason score (prostate cancer: *n* = 94, *P* < 0.001, unpaired *t*-test). **g** Overall survival analysis for patients with high and low expression of *AOC1* using the cutoff value.

Because it is known that *AOC1* has relatively low expression in prostate cancer, whether *AOC1* is associated with a poor prognosis for patients has attracted our attention. TCGA database data showed that low expression of *AOC1* is associated with a poor prognosis (Fig. 2a–c), thus confirming our hypothesis of significant ablation in metastatic prostate cancer (Fig. 2d). To further enhance the analysis, immunohistochemical experiments of the 94 specimens demonstrated that the expression of AOC1 gradually decreased with an increasing Gleason score (Fig. 2e, f). The Gleason score, T classification, lymph node metastasis, and distant metastasis are negatively correlated with AOC1 expression according to a clinical information analysis (Table 1), thus demonstrating its importance as a clinical indicator. Correspondingly, survival analysis showed that low expression of AOC1 is closely related to a poor prognosis (Fig. 2g, Supplementary Fig. 1e).

**Table 1:** Relationship between the expression of AOC1 in prostate cancer and clinic pathological parameters.

Characteristics	*N*	AOC1 IRS ≥ 10	AOC1 IRS < 10	*χ*^2^	*P*
*Age*					
≥70	64	14	50	0.344	0.558
<70	30	5	25		
*Gleason score*					
≥8	24	0	24	8.165	0.004
<8	70	19	51		
*T classification*					
T3, T4	54	4	50	12.903	<0.001
T1, T2	40	15	25		
*Lymph node metastasis*					
Present	36	3	33	5.105	0.024
Absent	58	16	42		
*Distant metastasis*					
Present	32	1	31	8.784	0.003
Absent	62	18	44		

### *AOC1* accretion depletes the malignancy of prostate cancer cells

**Fig. 3:**
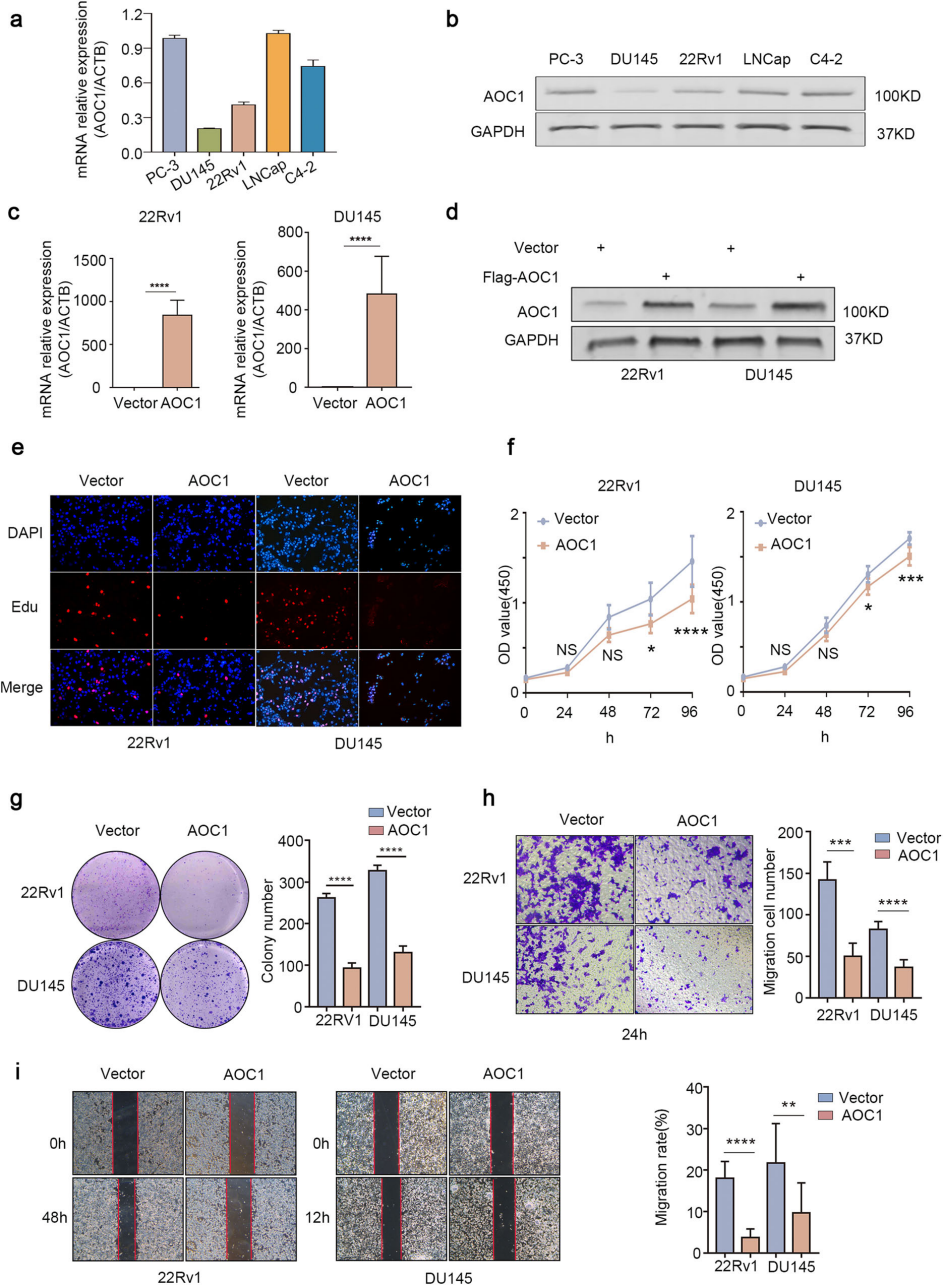
AOC1 accretion depletes the malignant potentials of prostate cancer cells. **a**, **b** RT-qPCR (**a**) and Western blot analysis (**b**) showed the expression of *AOC1* in 22Rv1 and DU145. GAPDH served as an internal reference. Unpaired *t*-test. **c**, **d**. RT-qPCR (**c**) and Western blot analysis (**d**) showed the efficiency of overexpressing *AOC1* in 22Rv1 and DU145. GAPDH served as an internal reference. Unpaired *t*-test. **e-g** CCK8 (**f**), EdU (**e**) and Colony Formation assays (**g**) showed the proliferation ability of 22Rv1 and DU145 after overexpression of *AOC1*. Unpaired *t*-test, ANOVA. **h**, **i** Wound-Healing (**i**) and Transwell Assay (**h**) showed the migration ability of 22Rv1 and DU145 after overexpression of *AOC1*. Unpaired *t*-test. (*P* < 0.05 as “*”; *P* < 0.01 as “**”; *P* < 0.001 as “***”).

To further explore the function of *AOC1*, we conducted a series of function studies of *AOC1*. Western blot and qRT-PCR verified that DU145 and 22Rv1 have relatively low expression of *AOC1* in all prostate cancer cell lines, but that LNCaP and PC-3 have the higher expression (Fig. 3a, b). To demonstrate the role of *AOC1* in the cell biology of prostate cancer, we overexpressed *AOC1* in prostate cancer cell lines 22Rv1 and DU145. The overexpression efficiency of *AOC1* was validated in both mRNA and protein levels (Fig. 3c, d). Consistent with clinical results, overexpression of *AOC1* is remarkably able to inhibit the growth of 22Rv1 and DU145 prostate cancer cells (Fig. 3f, g); however, the escalation of *AOC1* significantly decreased the EdU level in both 22Rv1 and DU145 cells (Fig. 3e, Supplementary Fig. 1f). The wound-healing assay (Fig. 3i) and Transwell assay (Fig. 3h), which reflect the migration ability, also showed that overexpression of *AOC1* inhibits the migration ability of both 22Rv1 and DU145 cells. To confirm our results, we knocked down *AOC1* in LNCaP and PC-3, which resulted in a significant increase in the malignant behaviors of prostate cancer cells after *AOC1* is knocked down (Supplementary Fig. 2a-i, Supplementary Fig. 3a, b). These findings demonstrated that *AOC1* supplementation can reduce the malignant capacity of prostate cancer cell lines.

### Spermidine supplements the *AOC1* anticancer effect

**Fig. 4:**
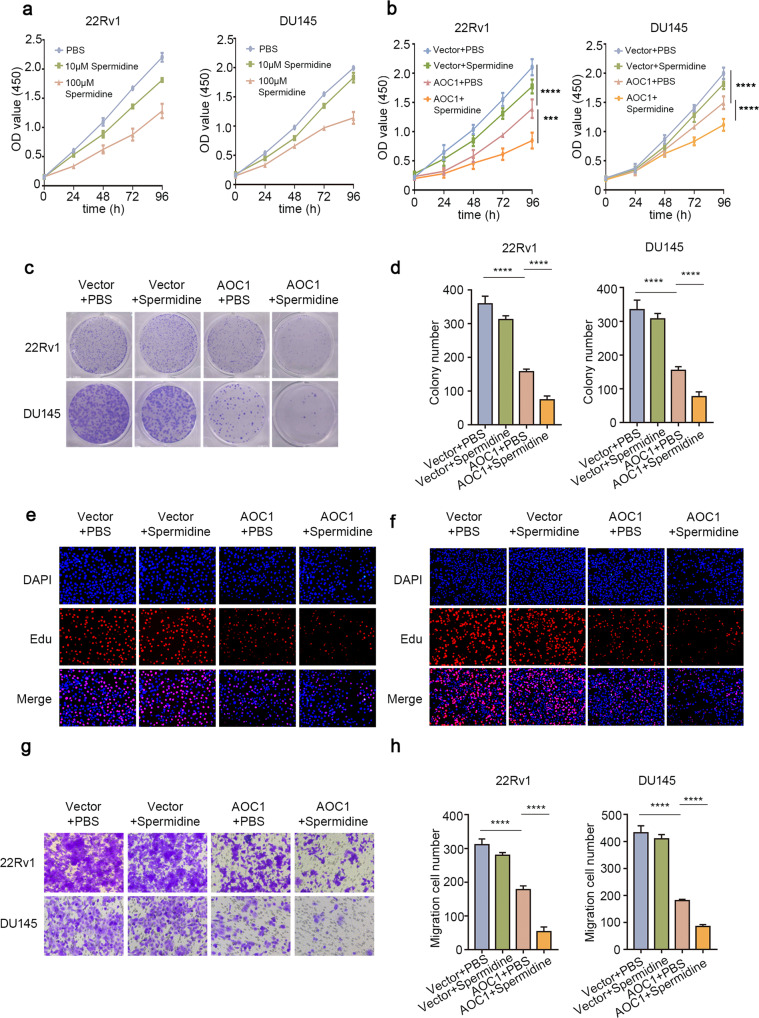
Spermidine supplements the anticancer effect of *AOC1*. **a** CCK8 assay showed the spermidine optimal concentration. **b–d** CCK8 (**b**), and Colony Formation assays (**c**, **d**) showed the proliferation ability of 22Rv1 and DU145 after overexpressing *AOC1* and adding spermidine. Unpaired *t*-test, ANOVA. **e**, **f** EdU showed the proliferation ability of 22Rv1 and DU145 after overexpressing *AOC1* and adding spermidine. Unpaired *t*-test. **g**, **h** Transwell Assay showed the migration ability of 22Rv1 and DU145 after overexpressing *AOC1* and adding spermidine. Unpaired *t*-test. (*P* < 0.05 as “*”; *P* < 0.01 as “**”; *P* < 0.001 as “***”).

Spermidine, as a polyamine, can be decomposed into ROS by AOC1. Therefore, we aimed to determine whether spermidine is the direct downstream function target that can regulate prostate cancer cell growth. First, we treated 22Rv1 and DU145 prostate cancer cells with different doses of spermidine. By performing preliminary experiments, we found that approximately 100 μM is the optimal effective concentration of spermidine (Fig. 4a, Supplementary Fig. 3c, d). We also confirmed that an appropriate concentration of spermidine can inhibit the proliferation of prostate cancer cell lines. Additionally, our results showed that low concentrations of spermidine (10 μM) could achieve better anti-cancer growth effects when accompanied by AOC1 overexpression (Fig. 4b-f, Supplementary Fig. 3e). Similarly, the Transwell assay also demonstrated that low concentrations of spermidine (10 μM) could be reinforced by AOC1 overexpression (Fig. 4g, h). All these results suggested that spermidine supplementation was able to significantly increase the inhibitory effect of AOC1 on tumor progression.

### ROS produced by AOC1 further triggers ferroptosis

**Fig. 5:**
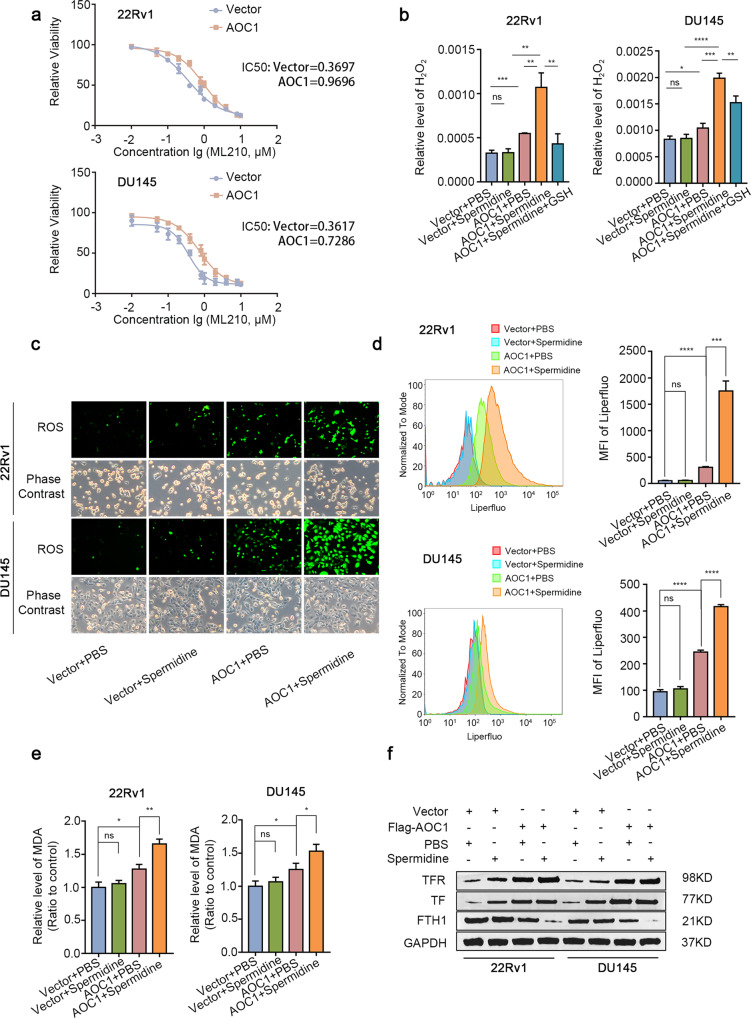
ROS produced by AOC1 further triggers ferroptosis. **a** IC_50_ curve showed that the sensitivity of prostate cancer cell lines to ferroptosis inducer, ML210 after overexpressing *AOC1*. **b** H_2_O_2_ assay showed that the content of H_2_O_2_ significantly after overexpressing *AOC1* and adding spermidine. Unpaired *t*-test. **c** ROS assay showed that the content of ROS after overexpressing *AOC1* and adding spermidine. Unpaired *t*-test. **d** Liperfluo assay showed that the content of LPO after overexpressing *AOC1* and adding spermidine. Unpaired *t*-test. **e** MDA assay showed MDA level after AOC1 overexpression with or without Spermidine treatment in prostate cancer cell lines. Unpaired *t*-test. **f** Western blot analysis showed that the ferroptosis-associated proteins changed (TFR, TF, FTH1) after transfecting with *AOC1* plasmids and adding Spermidine. GAPDH served as an internal reference. Unpaired *t*-test. (*P* < 0.05 as “*”; *P* < 0.01 as “**”; *P* < 0.001 as “***”).

It is known that AOC1 and spermidine can suppress prostate cancer proliferation, but the mechanism remains unclear. Based on the correlation between AOC1 and ROS, it would be beneficial to know whether *AOC1* also has a role in ferroptosis. We treated both control 22Rv1 and DU145 cells with *AOC1* overexpression with different doses of ML210, which induced ferroptosis. The sensitivity of prostate cancer cells to ferroptosis inducers, such as ML210, was surprisingly reduced after *AOC1* overexpression, which means that *AOC1* may affect ferroptosis in prostate cancer cells (Fig. 5a). ROS is known to induce lipid peroxidation, which accelerates ferroptosis. The H_2_O_2_ and ROS assays confirmed that overexpression of AOC1, along with spermidine supplementation, can produce more ROS (Fig. 5b, c, Supplementary Fig. 3f, g), consistent with previous studies [18–20]. Similarly, the MDA and Liperfluo assays verified that simultaneous supplementation of spermidine and AOC1 can easily cause lipid peroxidation in both 22Rv1 and DU145 prostate cancer cell lines (Fig. 5d, e). Western blot further verified the expression of three ferroptosis markers; the expression levels of TFR and TF, which are always positively correlated with ferroptosis, were increased with spermidine supplementation or AOC1 overexpression. In contract, FTH1, a protein negatively correlated with ferroptosis, is considerably attenuated when AOC1 is overexpressed (Fig. 5f). To prove whether endogenous *AOC1* is necessary for spermidine to promote ferroptosis, we knocked down *AOC1* in LNCaP and PC-3 cells while supplementing with the optimal dose of spermidine (100 μM). The results showed that the cancer-suppressing effect of spermidine was greatly reduced after knocking down *AOC1*, suggesting that *AOC1* has an irreplaceable role in the ability of spermidine to promote ferroptosis (Supplementary Fig. 4a–f). These results suggest that the augmentation of *AOC1* along with spermidine supplementation could trigger ferroptosis in prostate cancer cells, thereby suppressing their malignancy.

### SOX15 as a transcription factor favorably regulates the expression of *AOC1*

**Fig. 6:**
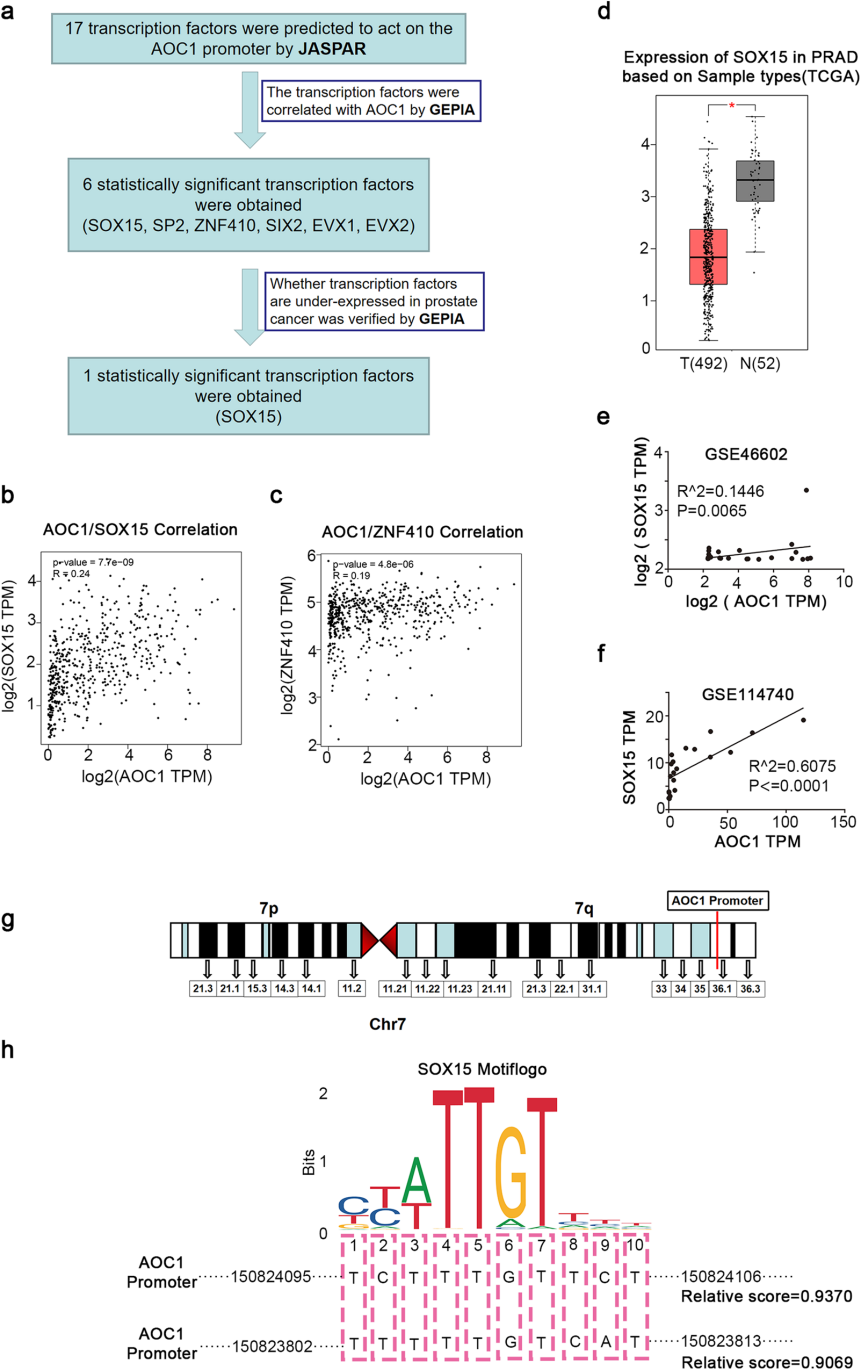
*SOX15*, as a transcription factor, favorably regulates the expression of *AOC1*. **a** The process of selecting the transcription factor *SOX15* upstream of *AOC1*. **b** The TCGA database showed there was a correlation between *SOX15* and *AOC1*. **c** The TCGA database showed the correlation between *ZNF410* and *AOC1*. **d** The TCGA database showed *SOX15* expression in prostate cancer. **e**, **f** The GEO datasets showed the correlation between *SOX15* and *AOC1* (GSE46602, GSE114740). **g** Location of *AOC1* promoter on the chromosome. **h** Diagram shows the binding site of SOX15 in the promoter of *AOC1*. (*P* < 0.05 as “*”; *P* < 0.01 as “**”; *P* < 0.001 as “***”).

Karin et al. reported Wilms tumor protein (WTI) activating transcription of the *AOC1* gene. However, WT1 expression is significantly increased in prostate cancer but is poorly correlated with *AOC1* in prostate cancer, which makes it difficult to explain why *AOC1* expression is significantly depleted in prostate cancer (Supplementary Fig. 5a, b). Therefore, there must be another mechanism that regulates *AOC1* expression in prostate cancer. We analyzed 17 potential transcription factors upstream of *AOC1* using the JASPAR database (Fig. 6a). Then, we immediately analyzed the association of each transcription factor with *AOC1* in the TCGA database (Fig. 6b, c, Supplementary Fig. 5c–f) and obtained six statistically significant transcription factors. We also performed cancerous and adjacent non-cancerous differential expression analyses of these six transcription factors using the TCGA database. The results showed that only the expression of SOX15 in prostate cancer was consistent with *AOC1* (Fig. 6d, Supplementary Fig. 5g–i). To further validate the correlation between SOX15 and *AOC1*, we performed a correlation analysis of *SOX15* and *AOC1* in GEO datasets GSE46602 and GSE114740. The results showed an obvious connection between *SOX15* and *AOC1* expression, which is consistent with our hypothesis (Fig. 6e, f). We excitedly identified a potential binding site of *AOC1* promoter on *SOX15*, which is located on chromosome 7 (Fig. 6g), and we identified two highly coincident binding motifs in the promoter of AOC1 (Fig. 6h) that are the potential binding sites of SOX15.

### SOX15 affects the progression of prostate cancer by regulating the expression of *AOC1*

**Fig. 7:**
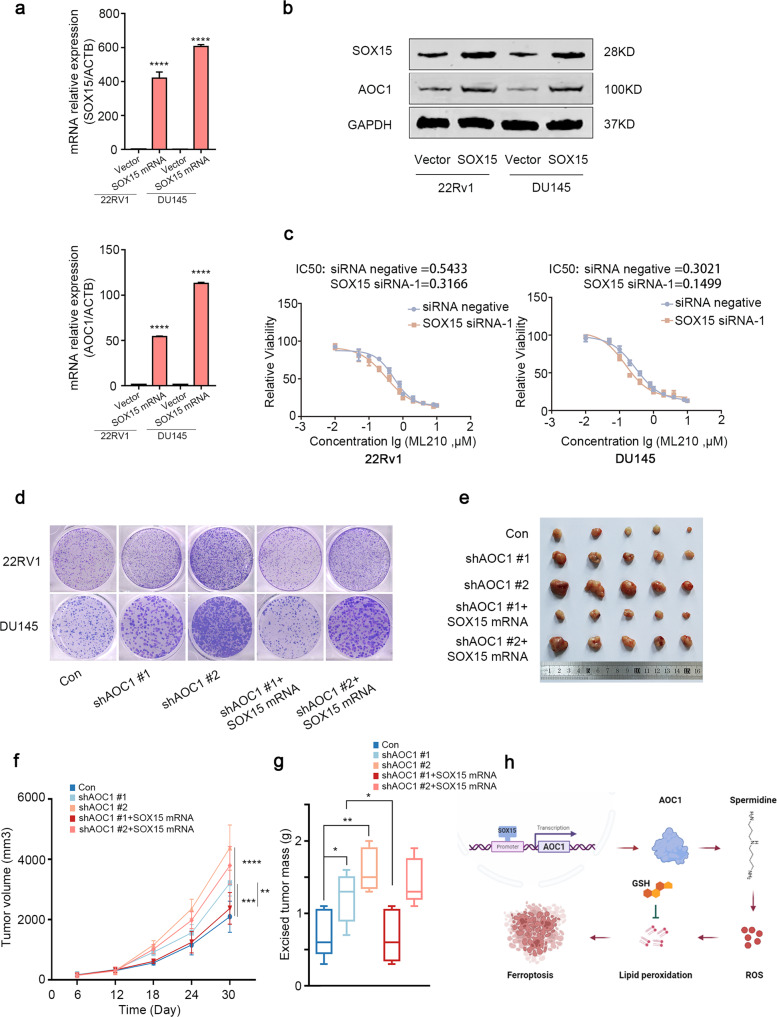
SOX15 affects the progression of prostate cancer by regulating the expression of *AOC1*. **a**, **b** RT-qPCR (**a**) and Western blot analysis (**b**) showed the expression of *SOX15* and *AOC1* in prostate cancer cell lines after overexpressing *SOX15*. GAPDH served as an internal reference. Unpaired *t*-test. **c** IC_50_ curve showed that the sensitivity of prostate cancer cells to ferroptosis inducer, ML210 after knocking down SOX15. **d** 22Rv1 and DU145 cells transfected with indicated shRNA or plasmid, and colony formation assay showed the proliferation ability of prostate cancer cells. **e–g** 22Rv1 cell transfected with indicated plasmid was injected into the right groin of SCID) mice, the tumors were collected after 33 days (**e**), the tumor volume (**f**) and tumor weight (**g**) were measured to show the tumor growth, Unpaired t-test, ANOVA. (*P* < 0.05 as “*”; *P* < 0.01 as “**”; *P* < 0.001 as “***”). **h** Specific mechanistic diagram of the SOX15/*AOC1*/ROS axis.

To confirm the regulation methods of *SOX15* on *AOC1*, we first knocked down the expression of *SOX15* by using different siRNAs and evaluated the expression of *SOX15* and *AOC1*. The results showed that knocking down *SOX15* will significantly decrease the *AOC1* expression in mRNA and protein, which demonstrated that *SOX15* could transcriptionally promote the expression of *AOC1* (Supplementary Fig. 6a, b). Simultaneously, we confirmed that the expression of *AOC1* was also upregulated after overexpression of *SOX15* (Fig. 7a, b). We also verified the sensitivity of two prostate cancer cell lines to ferroptosis inducer ML210 after knocking down SOX15. The results showed that the depletion of SOX15 will significantly decrease the IC_50_ of both 22Rv1 and DU145 cells to ML210 (Fig. 7c). Colony formation assays in vitro also verified that supplementing *SOX15* can enhance the cancer suppression effect of *AOC1* (Fig. 7d, Supplementary Fig. 6c). Additionally, supplementing SOX15 enhanced the effects of AOC1 in the promotion of ROS production (Supplementary Fig. 6d, e). At the same time, 22Rv1 cells with different treatments were inoculated in severe combined immunodeficiency mice, and the results showed that AOC1 depletion could significantly accelerate tumor growth in vivo (Fig. 7e–g). However, overexpressing *SOX15* will reverse the effect of AOC1 on tumor growth (Fig. 7e–g). In summary, AOC1 depletion was able to accelerate the progression of prostate cancer cells, which was inhibited after simultaneously overexpressing *SOX15*, further demonstrating the mechanism of action of the SOX15/*AOC1*/ferroptosis axis in the modulation of prostate cancer development.

## Discussion

For patients with recurrent prostate cancer, antiandrogen therapy is often the first choice, but most will have progression to CRPC [21]. How to treat CRPC and compensate for the lack of antiandrogen therapy have become great concerns for most urologists. The main purpose of our study was to explore a new target that may improve the prognosis of surgical patients and survival time for patients with CRPC [22, 23]. Three genes (*CD177*, *DUOX1*, and *AOC1*) were downregulated in prostate cancer through the analysis of bioinformatics data; the roles of both CD177 and DUOX1 in prostate cancer have been reported. Hence, we focused on the unstudied gene, *AOC1*.

*AOC1* catalyzes the degradation of spermine or spermidine. Studies have shown the diverse involvement of *AOC1* in allergic and immune responses, tumor formation, and tissue differentiation [24, 25]. *AOC1* can promote epithelial–mesenchymal transition via the AKT pathway in digestive system malignancies (gastric and colorectal cancer), thereby promoting tumor progression. Although the effect of *AOC1* on kidney development via WT1 has been reported [4], its role in prostate cancer remains unknown, but of great interest. We confirmed that supplementation of *AOC1* expression was able to inhibit the proliferation and migration of prostate cancer cell lines (22Rv1 and DU145), suggesting that *AOC1* acts as a prostate cancer tumor suppressor gene.

One of the substrates for AOC1, spermidine, has many biological functions, such as delaying aging and resisting inflammation [10]. In the prostate gland, spermidine has an important role [26, 27], and it has been demonstrated that both cancer tissue and blood spermidine levels are slightly increased in patients with prostate cancer [28]. We confirmed that artificial supplementation with large doses of spermidine prevented prostate cancer progression, which may be related to the dual effects of its H_2_O_2_ metabolites (ROS). In prostate cancer, moderate amounts of ROS promote tumor progression; however, excessive amounts of ROS inhibit tumor progression [29–31]. AOC1 was involved in the following processes as an enzyme:

$${{{\mathrm{R}}}}\!-\!{{{\mathrm{CH}}}}_2{{{\mathrm{NH}}}}_3^ + + {{{\mathrm{O}}}}_2 + {{{\mathrm{H}}}}_2{{{\mathrm{O}}}} \to {{{\mathrm{R}}}}\!-\!{{{\mathrm{CHO}}}} + {{{\mathrm{NH}}}}_4^ + + {{{\mathrm{H}}}}_2{{{\mathrm{O}}}}_2\left( {{{{\mathrm{ROS}}}}} \right)$$

which provided adequate ROS in the presence of sufficient spermidine, thus inhibiting tumor progression. Future studies should evaluate the role of H_2_O_2_ in the inhibition of tumor progression. During this study, we confirmed that ferroptosis has a key role that promotes tumor death and inhibits tumor progression [18].

Based on the results of the bioanalysis, we speculated that SOX15 can positively regulate *AOC1* expression. SOX (the sex-determining region Y-box gene) has irreplaceable roles in tumorigenesis and embryonic development [12, 32, 33]. As a member, SOX15 has acted as a tumor suppressor in the digestive and urinary systems [13, 34, 35]. We demonstrated that the transcription factor SOX15 could positively regulate *AOC1* expression, thus promoting the cancer inhibition effect of *AOC1*. This provides a new research direction regarding the effects of *AOC1* and *SOX15* (Fig. 7h).

Few studies have evaluated the protective effect of ferroptosis on prostate cancer patients. We propose that the progression of prostate cancer can be inhibited by inducing ferroptosis in prostate cancer cells, which may become an innovative CRPC treatment. However, medical research cannot be limited to cell experiments. Safe and feasible clinical trials are needed to confirm its role in the human body. We hope that our research results can be promptly applied for the benefit of more patients.

## Conclusions

This study innovatively demonstrates that the transcription factor SOX15 can positively regulate *AOC1* expression in prostate cancer, whereas ROS generated by the breakdown of spermidine by *AOC1* can promote ferroptosis, thus playing an important role in inhibiting prostate cancer progression. The SOX15/*AOC1*/ROS axis provides new research directions for the treatment of CRPC.

## Supplementary information

Supplementary Information

Original Data File

Checklist

## Data Availability

All data supporting the findings of this study are available within the article, the Supporting Information files, or the corresponding author upon request.

## References

[1] Barbry P, Champe M, Chassande O, Munemitsu S, Champigny G, Lingueglia E (1990). Human kidney amiloride-binding protein: cDNA structure and functional expression. Proc Natl Acad Sci USA.

[2] Chassande O, Renard S, Barbry P, Lazdunski M (1994). The human gene for diamine oxidase, an amiloride binding protein. Molecular cloning, sequencing, and characterization of the promoter. J Biol Chem.

[3] Lopes de Carvalho L, Bligt-Linden E, Ramaiah A, Johnson MS, Salminen TA (2019). Evolution and functional classification of mammalian copper amine oxidases. Mol Phylogenet Evol.

[4] Kirschner KM, Braun JF, Jacobi CL, Rudigier LJ, Persson AB, Scholz H (2014). Amine oxidase copper-containing 1 (AOC1) is a downstream target gene of the Wilms tumor protein, WT1, during kidney development. J Biol Chem.

[5] Liu F, Ou W, Tang W, Huang Z, Zhu Z, Ding W (2021). Increased AOC1 expression promotes cancer progression in colorectal cancer. Front Oncol.

[6] Xu F, Xu Y, Xiong JH, Zhang JH, Wu J, Luo J (2020). AOC1 contributes to tumor progression by promoting the AKT and EMT pathways in gastric cancer. Cancer Manag Res.

[7] Fan J, Feng Z, Chen N (2020). Spermidine as a target for cancer therapy. Pharmacol Res.

[8] Porter CW, Bergeron RJ (1983). Spermidine requirement for cell proliferation in eukaryotic cells: structural specificity and quantitation. Science.

[9] Eisenberg T, Abdellatif M, Zimmermann A, Schroeder S, Pendl T, Harger A (2017). Dietary spermidine for lowering high blood pressure. Autophagy.

[10] Madeo F, Eisenberg T, Pietrocola F, Kroemer G. Spermidine in health and disease. Science 2018;359. 10.1126/science.aan2788 29371440

[11] Zhang D, Guo S, Wang H, Hu Y (2020). SOX15 exerts antitumor function in glioma by inhibiting cell proliferation and invasion via downregulation of Wnt/beta-catenin signaling. Life Sci.

[12] Moradi A, Ghasemi F, Anvari K, Hassanian SM, Simab SA, Ebrahimi S (2017). The cross-regulation between SOX15 and Wnt signaling pathway. J Cell Physiol.

[13] Wang S, Yang H, Chen X, Jiang Z (2018). Effects of SOX15 on the colorectal cancer cells via downregulation of the Wnt/beta-catenin signaling pathway. Future Oncol.

[14] Long MD, Singh PK, Russell JR, Llimos G, Rosario S, Rizvi A (2019). The miR-96 and RARgamma signaling axis governs androgen signaling and prostate cancer progression. Oncogene.

[15] Stockwell BR, Jiang X, Gu W (2020). Emerging mechanisms and disease relevance of ferroptosis. Trends Cell Biol.

[16] Zheng J, Conrad M (2020). The metabolic underpinnings of ferroptosis. Cell Metab.

[17] Wang S, Liu W, Wang J, Bai X (2020). Curculigoside inhibits ferroptosis in ulcerative colitis through the induction of GPX4. Life Sci.

[18] Dawkes HC, Phillips SE (2001). Copper amine oxidase: cunning cofactor and controversial copper. Curr Opin Struct Biol.

[19] Xie Y, Hou W, Song X, Yu Y, Huang J, Sun X (2016). Ferroptosis: process and function. Cell Death Differ.

[20] Yang WS, Stockwell BR (2016). Ferroptosis: death by lipid peroxidation. Trends Cell Biol.

[21] Dunning NL, Laversin SA, Miles AK, Rees RC (2011). Immunotherapy of prostate cancer: should we be targeting stem cells and EMT?. Cancer Immunol, Immunother.

[22] Teo MY, Rathkopf DE, Kantoff P (2019). Treatment of advanced prostate cancer. Annu Rev Med.

[23] Ku SY, Gleave ME, Beltran H (2019). Towards precision oncology in advanced prostate cancer. Nat Rev Urol.

[24] Stenzel I, Otto M, Delker C, Kirmse N, Schmidt D, Miersch O (2012). ALLENE OXIDE CYCLASE (AOC) gene family members of *Arabidopsis thaliana*: tissue- and organ-specific promoter activities and in vivo heteromerization. J Exp Bot.

[25] McGrath AP, Hilmer KM, Collyer CA, Shepard EM, Elmore BO, Brown DE (2009). Structure and inhibition of human diamine oxidase. Biochemistry.

[26] Affronti HC, Rowsam AM, Pellerite AJ, Rosario SR, Long MD, Jacobi JJ (2020). Pharmacological polyamine catabolism upregulation with methionine salvage pathway inhibition as an effective prostate cancer therapy. Nat Commun.

[27] Basu HS, Thompson TA, Church DR, Clower CC, Mehraein-Ghomi F, Amlong CA (2009). A small molecule polyamine oxidase inhibitor blocks androgen-induced oxidative stress and delays prostate cancer progression in the transgenic adenocarcinoma of the mouse prostate model. Cancer Res.

[28] Chaisiri P, Harper ME, Blamey RW, Peeling WB, Griffiths K (1980). Plasma spermidine concentrations in patients with tumours of the breast or prostate or testis. Clin Chim Acta.

[29] Wang Y, Zhang Y, Ru Z, Song W, Chen L, Ma H (2019). A ROS-responsive polymeric prodrug nanosystem with self-amplified drug release for PSMA (-) prostate cancer specific therapy. J Nanobiotechnol.

[30] Mehraein-Ghomi F, Basu HS, Church DR, Hoffmann FM, Wilding G (2010). Androgen receptor requires JunD as a coactivator to switch on an oxidative stress generation pathway in prostate cancer cells. Cancer Res.

[31] Schieber M, Chandel NS (2014). ROS function in redox signaling and oxidative stress. Curr Biol.

[32] Li L, Li L, Li Q, Liu X, Ma X, Yong J (2021). Dissecting the epigenomic dynamics of human fetal germ cell development at single-cell resolution. Cell Res.

[33] Maruyama M, Ichisaka T, Nakagawa M, Yamanaka S (2005). Differential roles for Sox15 and Sox2 in transcriptional control in mouse embryonic stem cells. J Biol Chem.

[34] Thu KL, Radulovich N, Becker-Santos DD, Pikor LA, Pusic A, Lockwood WW (2014). SOX15 is a candidate tumor suppressor in pancreatic cancer with a potential role in Wnt/beta-catenin signaling. Oncogene.

[35] Yan HT, Shinka T, Sato Y, Yang XJ, Chen G, Sakamoto K (2007). Overexpression of SOX15 inhibits proliferation of NT2/D1 cells derived from a testicular embryonal cell carcinoma. Mol Cells.

