# Supplementary material for: SOX15 transcriptionally increases the function of AOC1 to modulate ferroptosis and progression in prostate cancer
Source: Cell Death Dis. 2022 Aug 3;13(8):673. doi: 10.1038/s41419-022-05108-w (PMC9349193; doi:10.1038/s41419-022-05108-w)
Supplement: Supplementary file 1 — Supplementary Information [file 41419_2022_5108_MOESM1_ESM.docx]

**SOX15 transcriptionally increases the function of *AOC1* to modulate ferroptosis and progression in prostate cancer**

*Yinghui Ding, Yuankang Feng, Zhenlin Huang, Yu Zhang, Xiang Li, Ruoyang Liu, Hao Li, Tao Wang, Yafei Ding, Zhankui Jia, Jinjian Yang*

**Files included in supplementary information**

**Supplementary Fig 1 （Supplemental to Fig. 1-3）**

**Supplementary Fig 2 （Supplemental to Fig. 3）**

**Supplementary Fig 3 （Supplemental to Fig. 3-5）**

**Supplementary Fig 4 （Supplemental to Fig. 5）**

**Supplementary Fig 5 （Supplemental to Fig. 6）**

**Supplementary Fig 6 （Supplemental to Fig. 7）**

**Supplementary Table 1. Oligonucleotides used for relative gene expression by qRT-PCR**

**Supplementary Table 2. The oligonucleotides of si-SOX15 or sh-SOX15.**


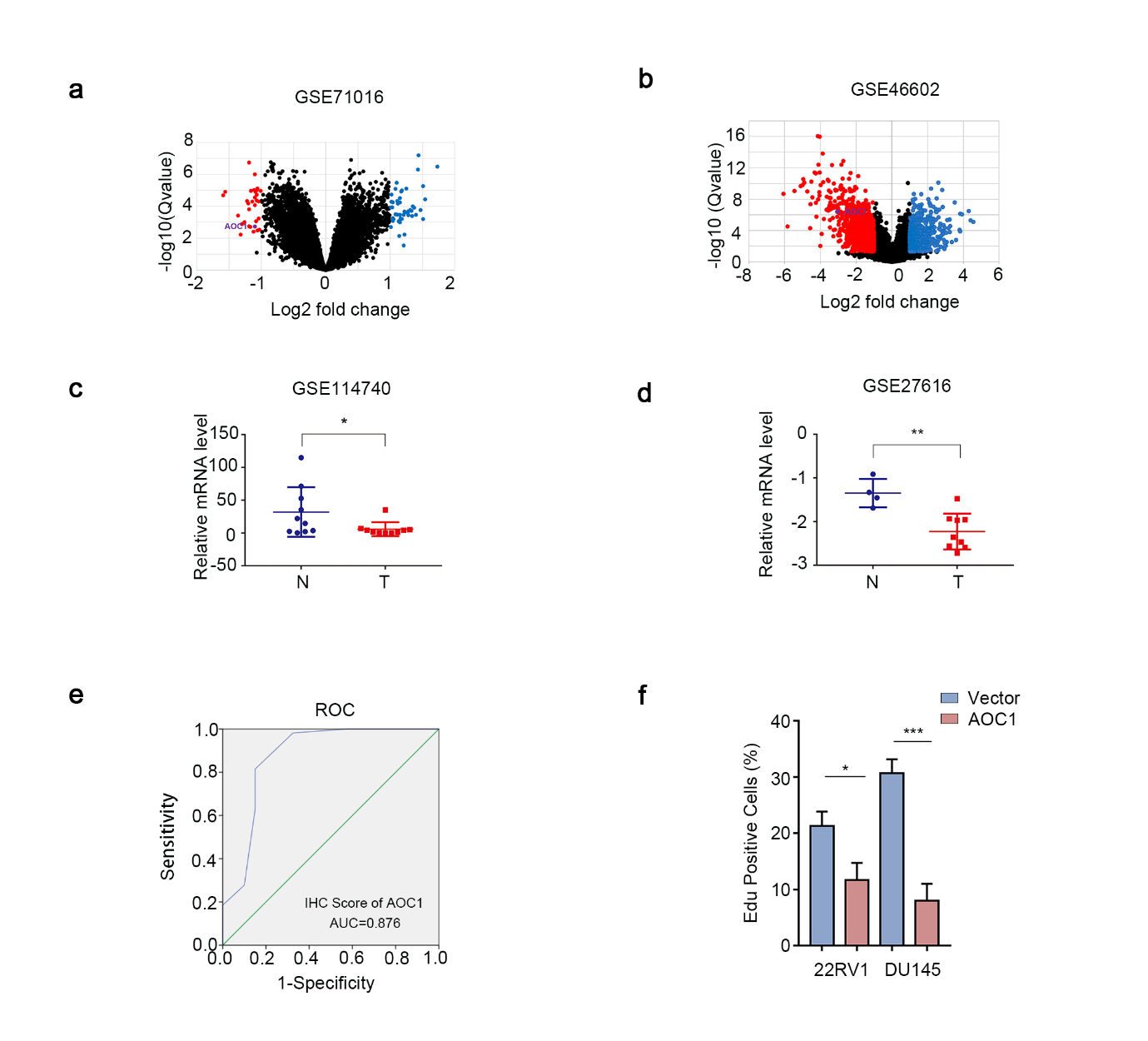


**Supplementary Fig 1.**

**a**, **b** Volcano plot demonstrating differentially expressed genes between prostate cancer and normal tissues based on GEO datasets GSE 71016 and GSE46602. **c**, **d** *AOC1* expression in prostate cancer based on GEO datasets (GSE114740 and GSE 27616). **e** The cut-off value was found out using the ROC curve of AOC1 staining. **f** Quantification of EdU positivity (%) showed the proliferation ability of 22Rv1 and DU145 was significantly limited after overexpression of *AOC1*. Unpaired t test. (*P* < 0.05 as “*”; *P< 0.01* as “**”; *P< 0.001* as “***”)


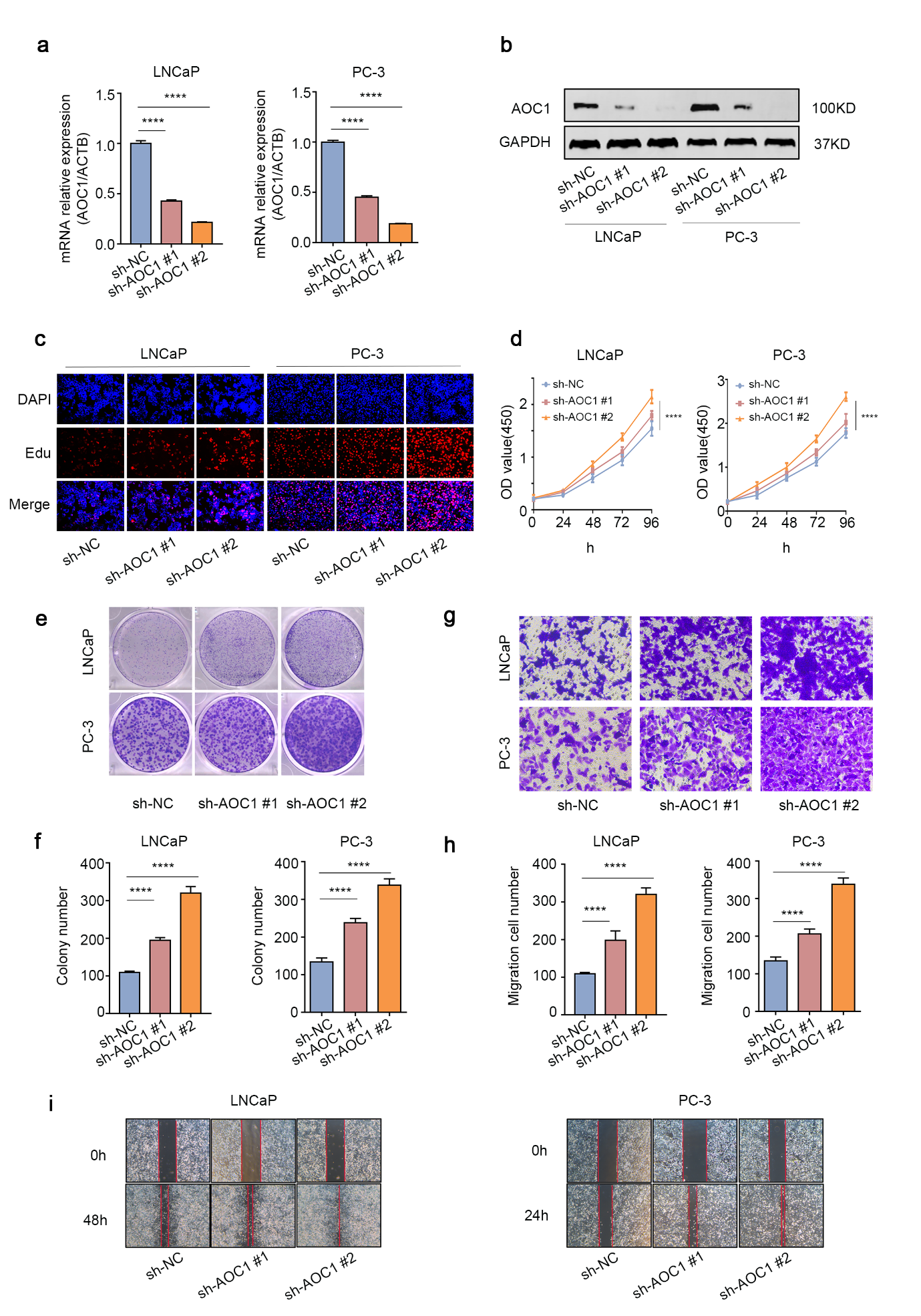


**Supplementary Fig 2.**

**a**, **b** RT-qPCR (**a**) and Western blot analysis (**b**) showed the efficiency of silencing *AOC1* in LNCaP and PC-3. GAPDH served as an internal reference. Unpaired t test. **c-f** CCK8 (**d**), EdU (**c**) and Colony Formation assays (**e, f**) showed the proliferation ability of LNCaP and PC-3 after silencing *AOC1*. Unpaired t test, ANOVA. **g-i** Wound-Healing (**i**) and Transwell Assay (**g, h**) showed the migration ability of LNCaP and PC-3 after silencing *AOC1*. Unpaired t test. (*P*< 0.05 as “*”; *P< 0.01* as “**”; *P< 0.001* as “***”).


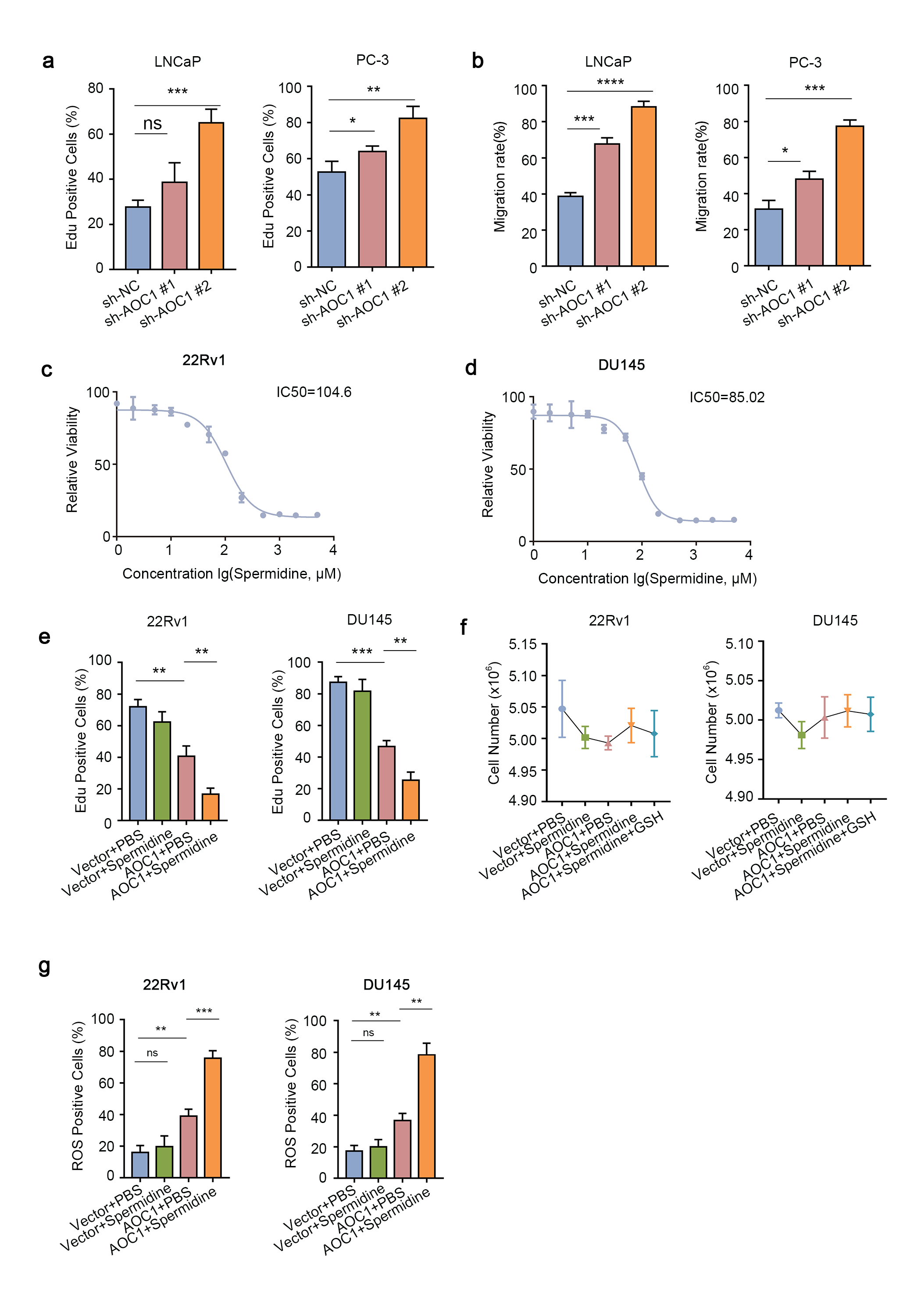


**Supplementary Fig 3.**

**a** Quantification of EdU positivity (%) showed the proliferation ability of LNCaP and PC-3 after silencing *AOC1*. Unpaired t test. **b** Quantification of migration rate (%) showed the migration ability of LNCaP and PC-3 after silencing *AOC1*. Unpaired t test. **c, d** IC50 curve shows sensitivity of prostate cancer cells to spermidine with CCK8 Assay. **e** Quantification of EdU positivity (%) showed the proliferation ability of 22Rv1 and DU145 after overexpressing AOC1 and adding spermidine. Unpaired t test. **f** The number of cells in H2O2 assay after overexpressing *AOC1* and spermidine treatment. **g** Quantification of ROS positivity (%) showed that the content of ROS after overexpressing *AOC1* and adding spermidine. Unpaired t test. (*P*< 0.05 as “*”; *P< 0.01* as “**”; *P< 0.001* as “***”).


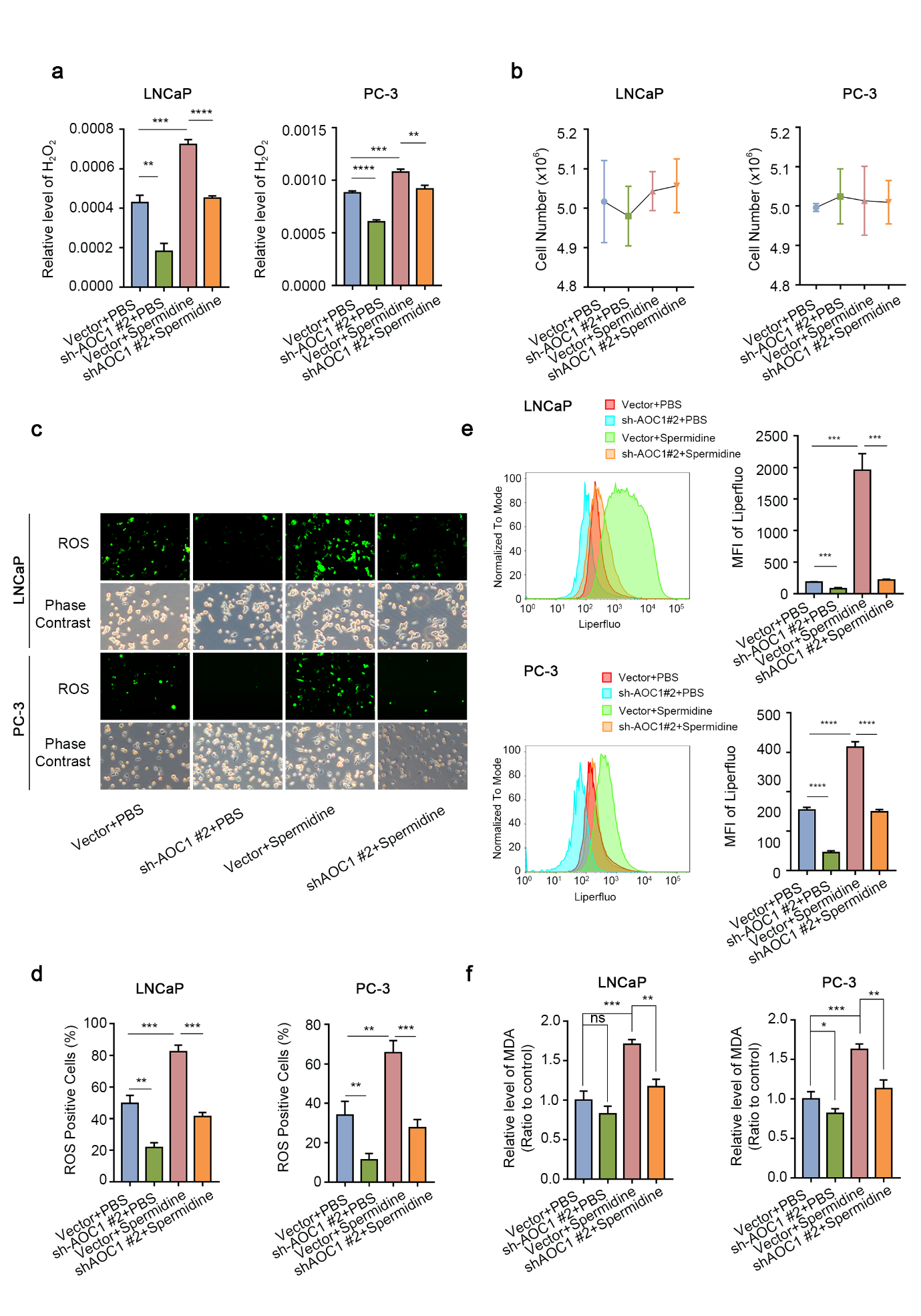


**Supplementary Fig 4.**

**a, b** H2O2 assay showed the content of H2O2 after silencing *AOC1* and adding spermidine. Unpaired t test. **c, d** ROS assay showed the content of ROS after silencing *AOC1* and spermidine treatment. Unpaired t test. **e** Liperfluo assay showed the content of LPO after silencing *AOC1* and spermidine treatment. Unpaired t test. **f** MDA assay showed MDA level after knocking down *AOC1* with or without Spermidine treatment in prostate cancer cell lines. Unpaired t test. (*P* < 0.05 as “*”; *P< 0.01* as “**”; *P< 0.001* as “***”)


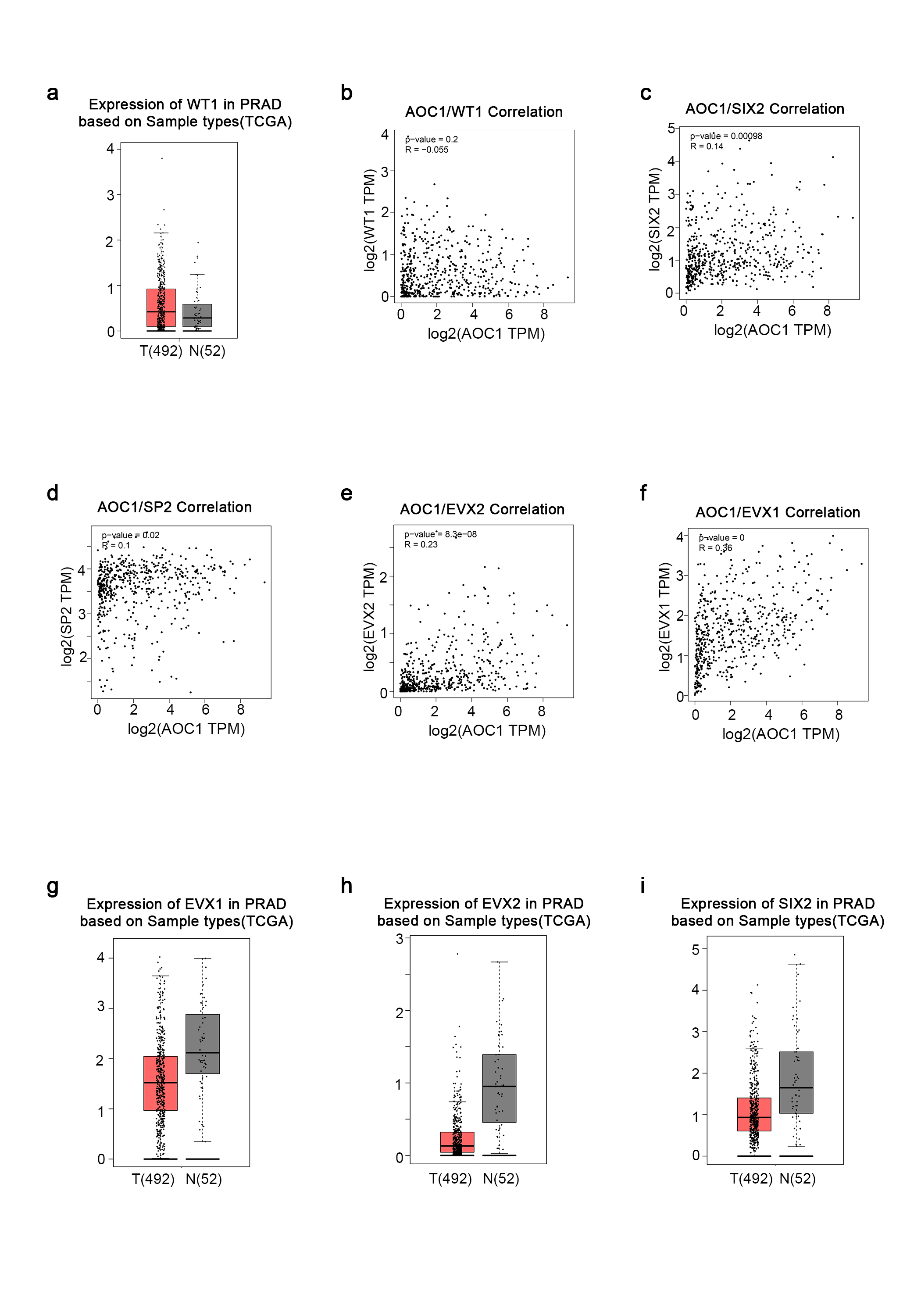


**Supplementary Fig 5.**

**a** WT1 expression on TCGA database. **b** Correlation analysis between *WT1* and *AOC1* based on TCGA database. **c-f** Correlation analysis between transcription factors (EVX1, EVX2, SIX2 and SP2) and *AOC1* based on TCGA database. **g-i** EVX1, EVX2, SIX2 expressions in prostate cancer based on TCGA database.


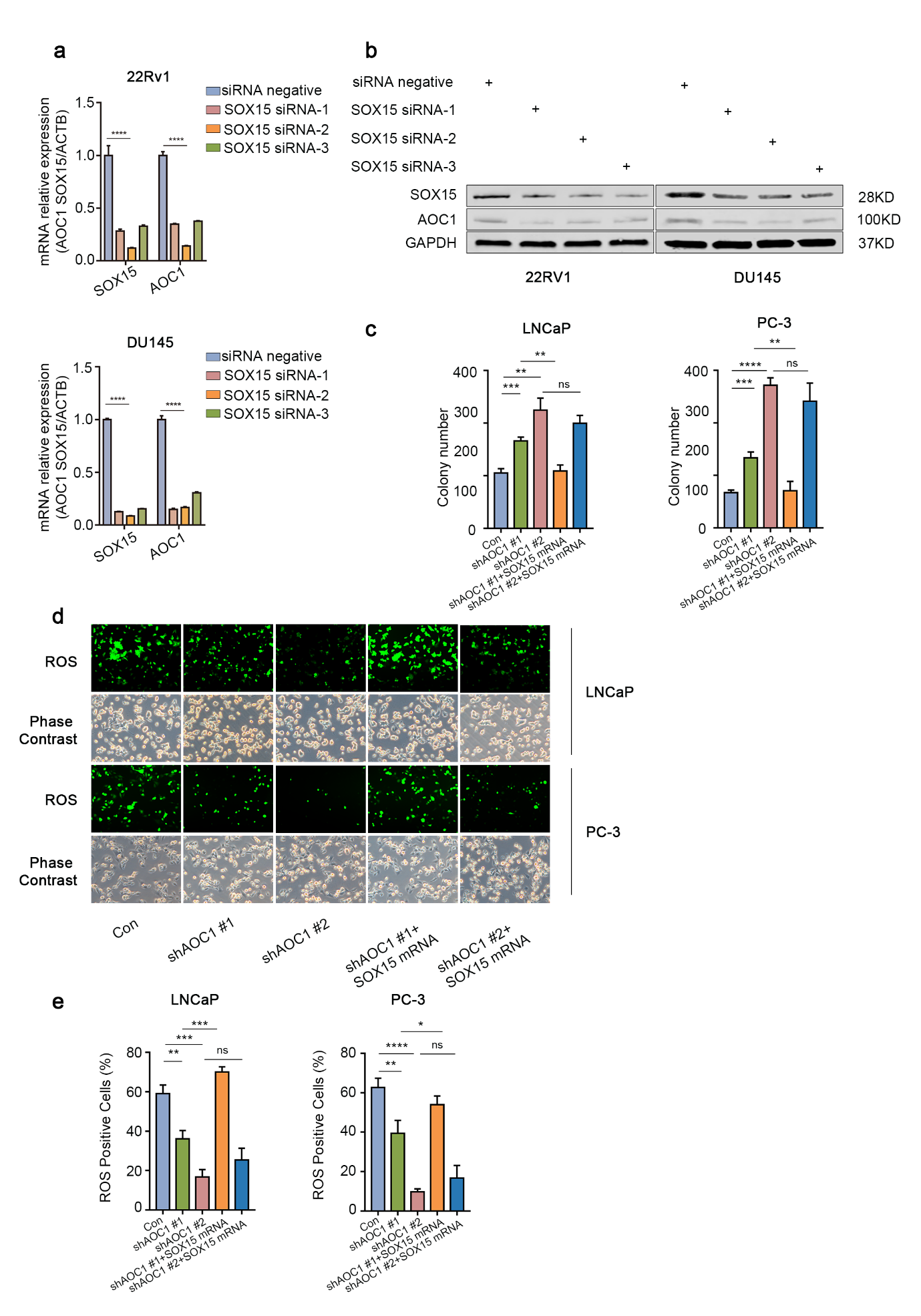


**Supplementary Fig 6.**

**a, b** RT-qPCR (**a**) and Western blot analysis (**b**) showed the expression of *SOX15* and *AOC1* in prostate cancer cell lines after knocking down SOX15. GAPDH served as an internal reference. Unpaired t test. **c** LNCaP and PC-3 cells transfected with indicated shRNA or plasmid, and quantification of colony number showed the proliferation ability of prostate cancer cells. Unpaired t test. **d, e** LNCaP and PC-3 cells transfected with indicated shRNA or plasmid, and ROS assay showed the ROS content of prostate cancer cells. Unpaired t test. (*P* < 0.05 as “*”; *P< 0.01* as “**”; *P< 0.001* as “***”)

**Supplementary Table 1. Oligonucleotides used for relative gene expression by qRT-PCR.**

| Target gene | Forward primers (5’-3’) | Reverse primers (5’-3’) |
| --- | --- | --- |
| AOC1 | ATTGCCTATGAGGTCAGCGTG | CAGCCGACATCGAGGTACTTG |
| SOX15 | GACTACCCCGACTACAAGTAC | GTCTGTACCCAAAGCCTCTG |
| ACTB | CTCCATCCTGGCCTCGCTGT | GCTGTCACCTTCACCGTTCC |

**Supplementary Table 2.** **The oligonucleotides of siRNA or shRNA.**

| Product name | Forward primers (5’-3’) |
| --- | --- |
| SOX15 siRNA-1 | GAACTGCTGCCCACCTATA* |
| SOX15 siRNA-2 | GCTCTCCCACTCCATACAA* |
| SOX15 siRNA-3 | AGCAGAGGCTTTGGGTACA* |
| SOX15 shRNA-1  SOX15 shRNA-2  AOC1 shRNA-1  AOC1 shRNA-2 | GGGAACTGCTGCCCACCTATA  GGAACTGCTGCCCACCTATAC  GCTCTGTGTGCTGCTTCTTGC  GCACAGGGCCATGTGTGTAGG |

* The oligonucleotides from RiboBio, Guangzhou, China.
