# Supplementary material for: SOX15 transcriptionally increases the function of AOC1 to modulate ferroptosis and progression in prostate cancer
Source: Cell Death Dis. 2022 Aug 3;13(8):673. doi: 10.1038/s41419-022-05108-w (PMC9349193; doi:10.1038/s41419-022-05108-w)
Supplement: Supplementary file 2 — Original Data File [file 41419_2022_5108_MOESM2_ESM.pdf]

Figure 1

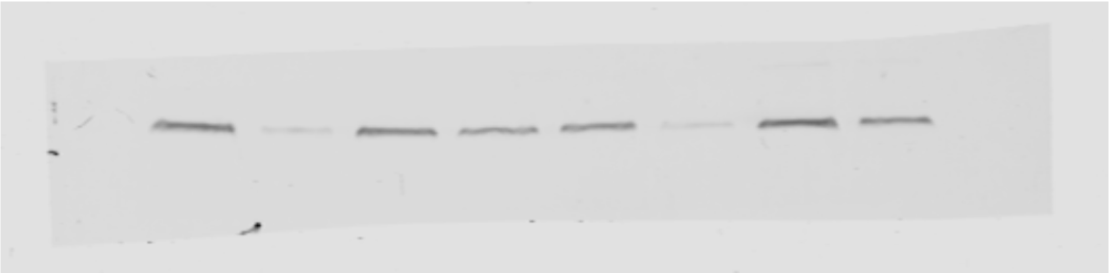

Patient 1-4 AOC1

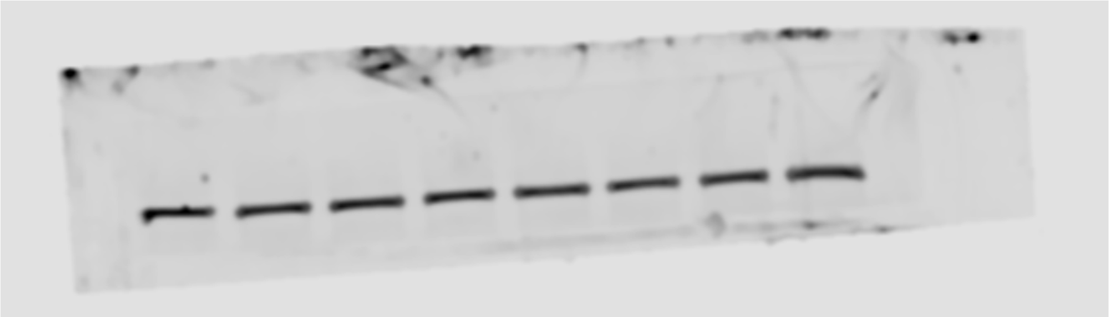

Patient 1-4 GAPDH

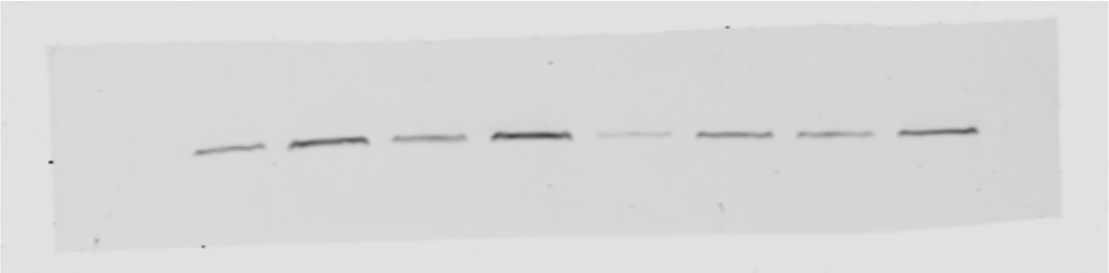

Patient 5-8 AOC1

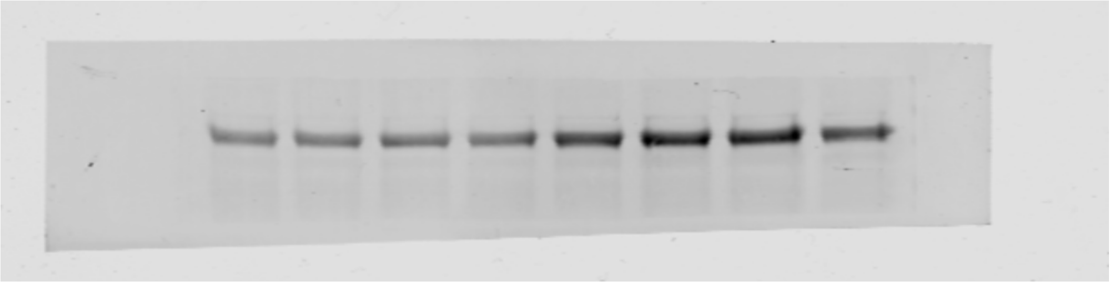

Patient 5-8 GAPDH

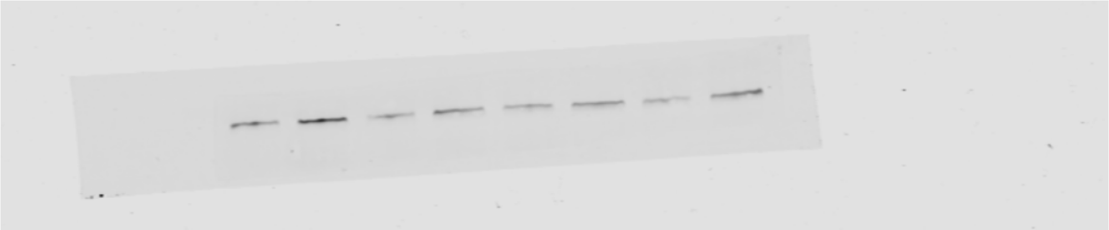
Patient 9-12 AOC1

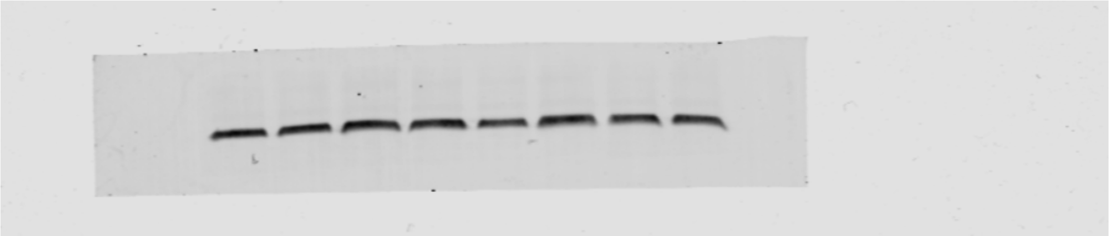
Patient 9-12 GAPDH

Figure 3

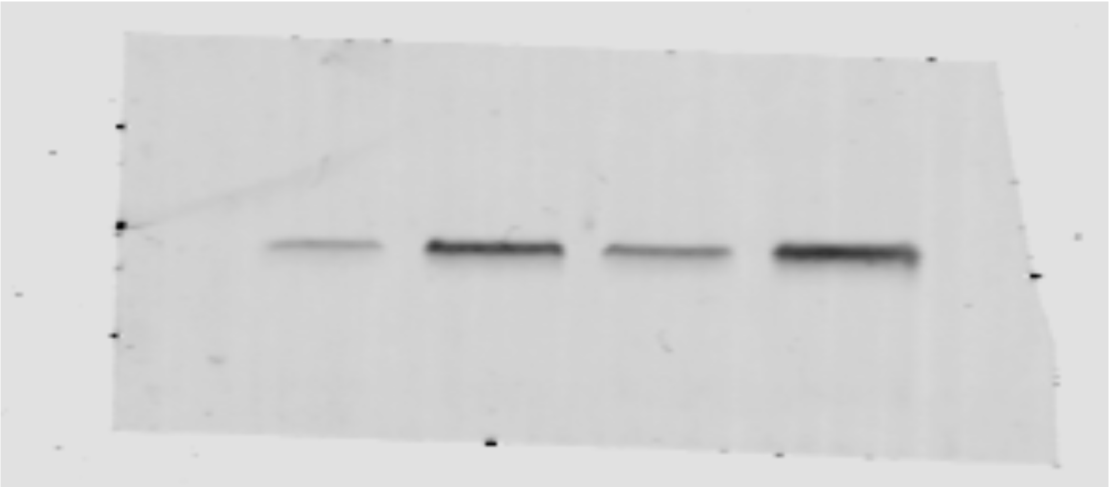

22Rv1 DU145 AOC1

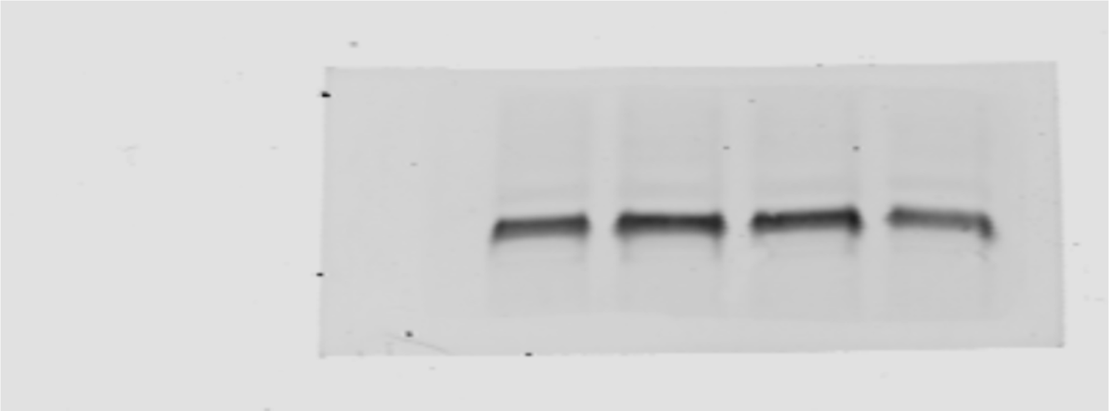

22Rv1 DU145 GAPDH

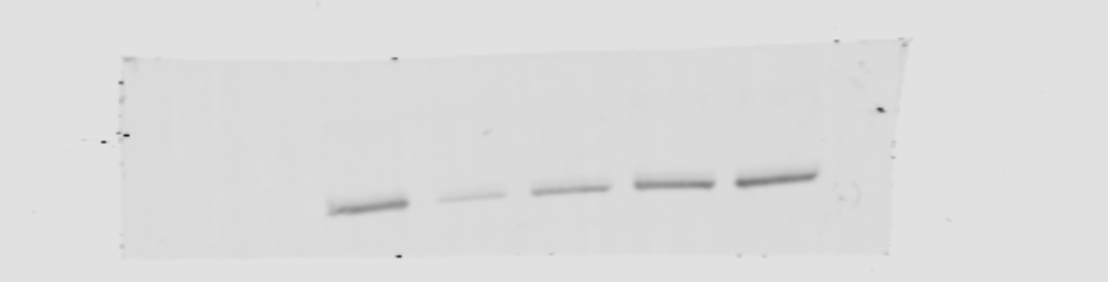

PC-3 DU145 22Rv1 lLNCaP C4-2 AOC1

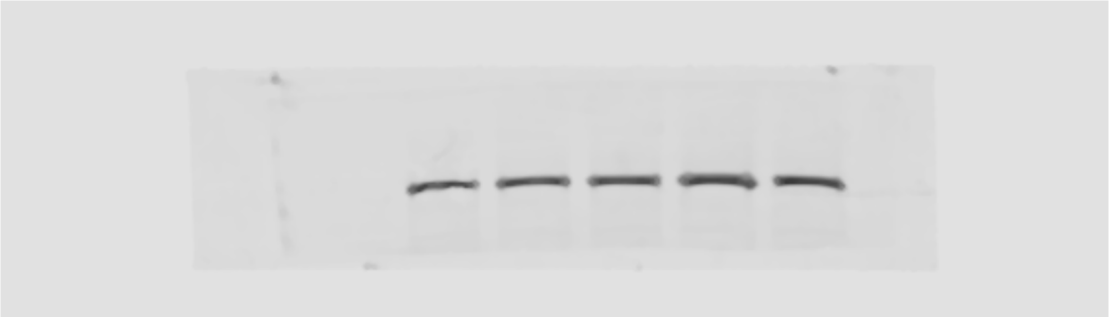

PC-3 DU145 22Rv1 lLNCaP C4-2 GAPDH

Figure 5

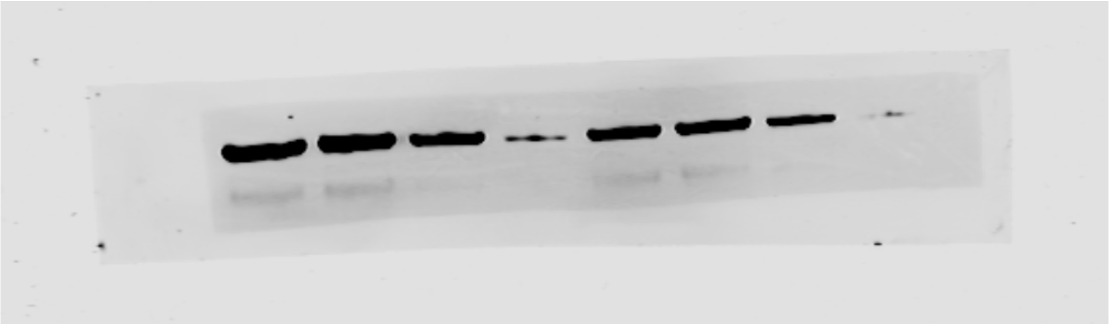

FTH1

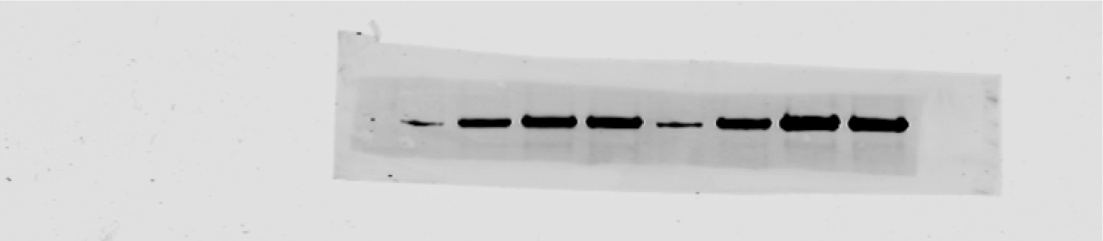

TF

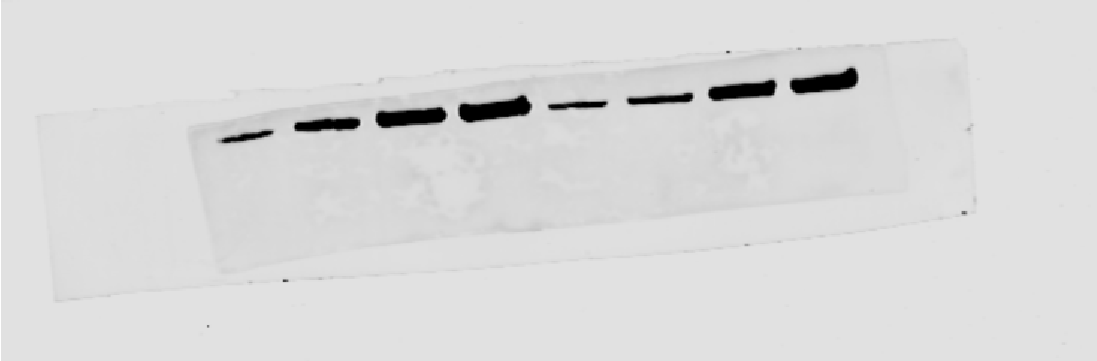

TFR

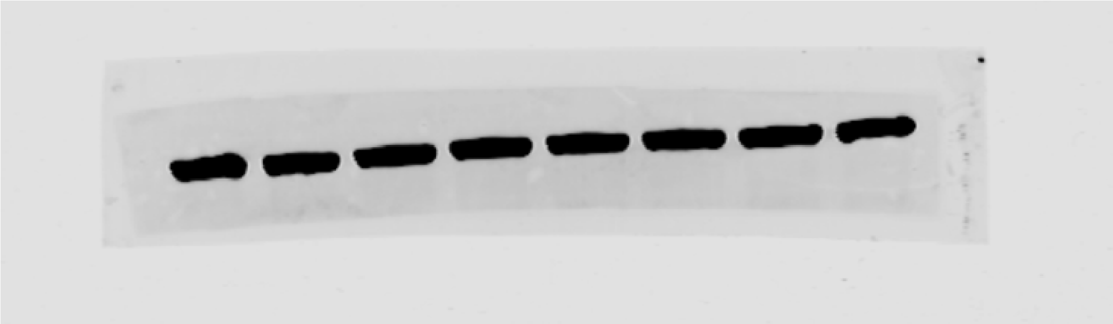

GAPDH

Figure 7

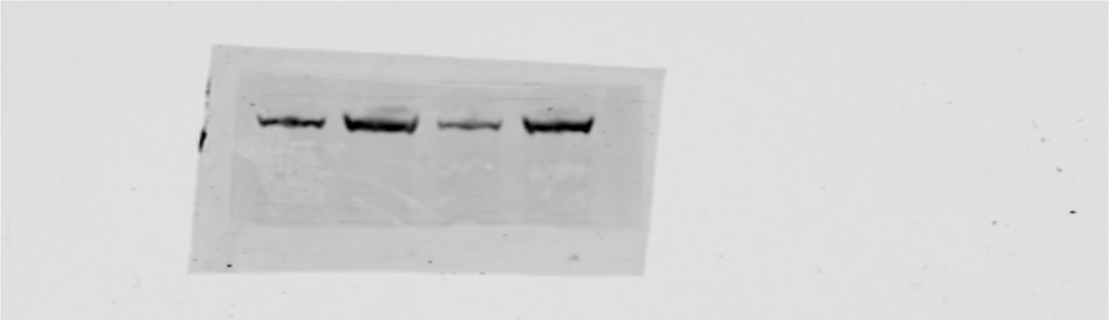

AOC1

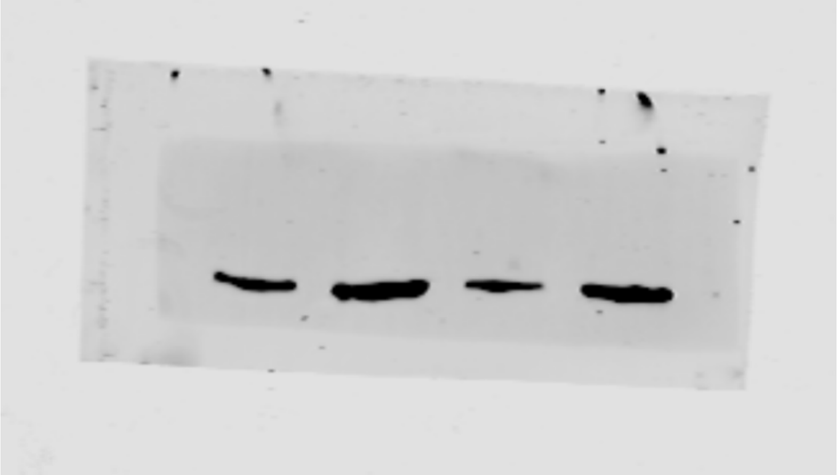

SOX15

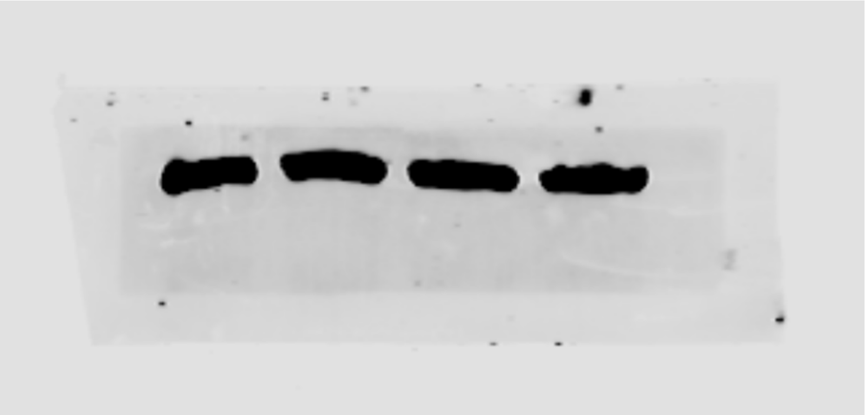

GAPDH

Supplementary Figure 2

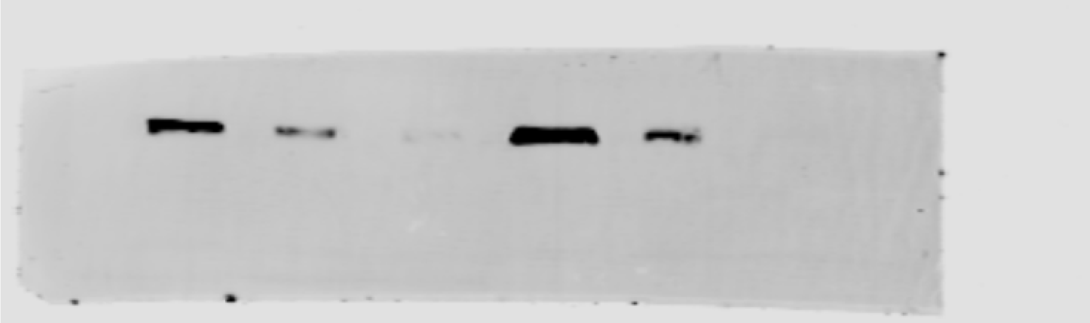

AOC1

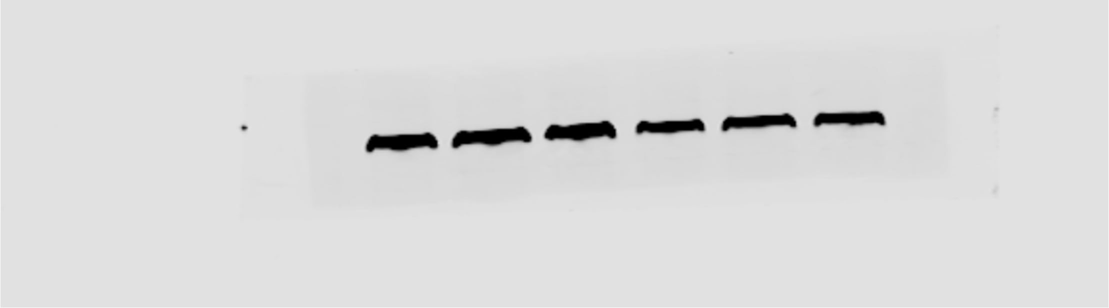

GAPDH

Supplementary Figure 6

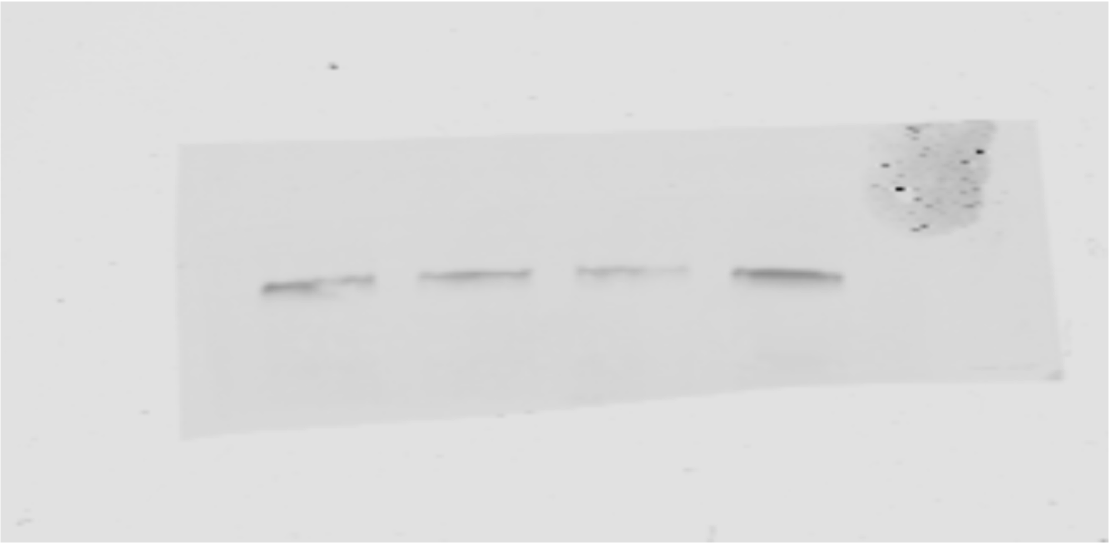

22Rv1 AOC1

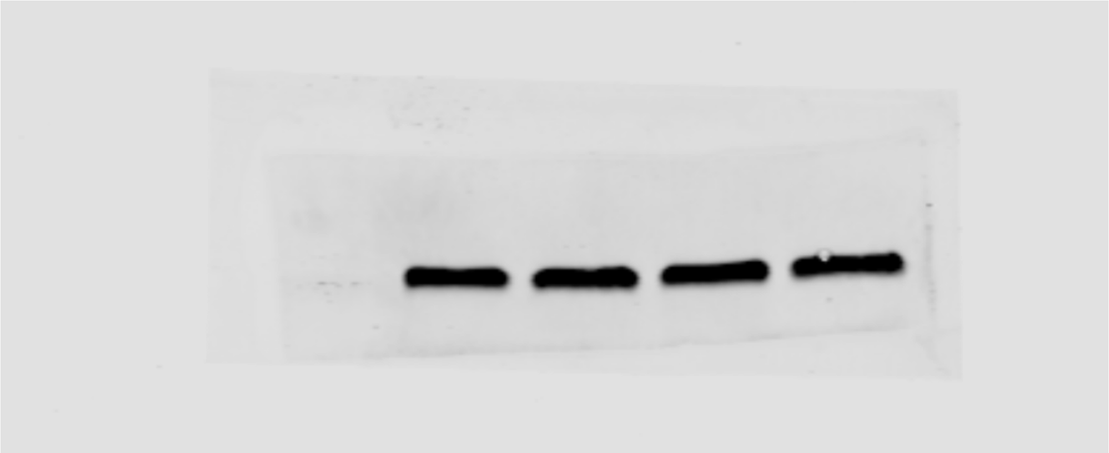

22Rv1 GAPDH

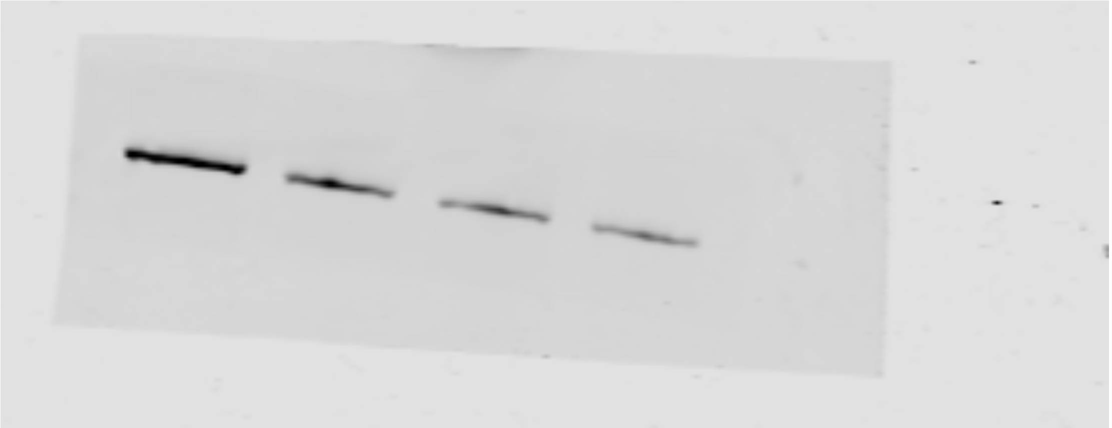

22Rv1 SOX15

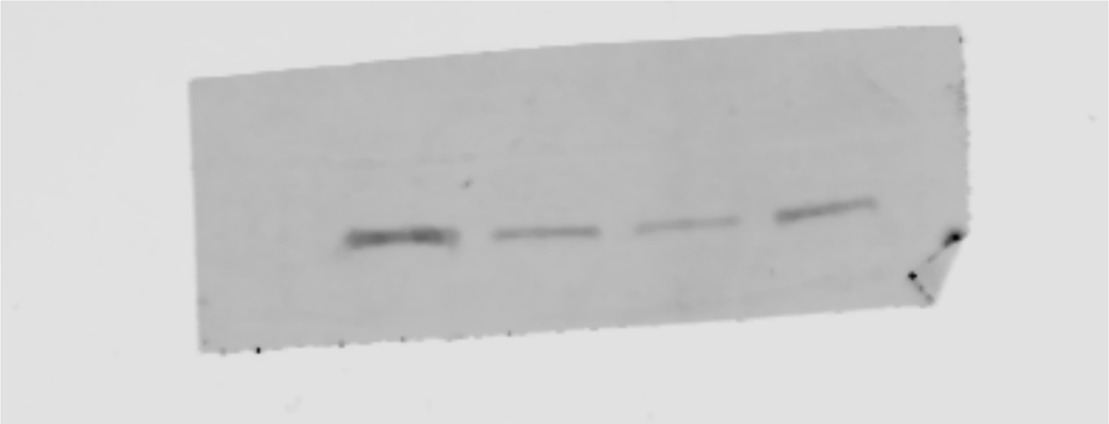

DU145 AOC1

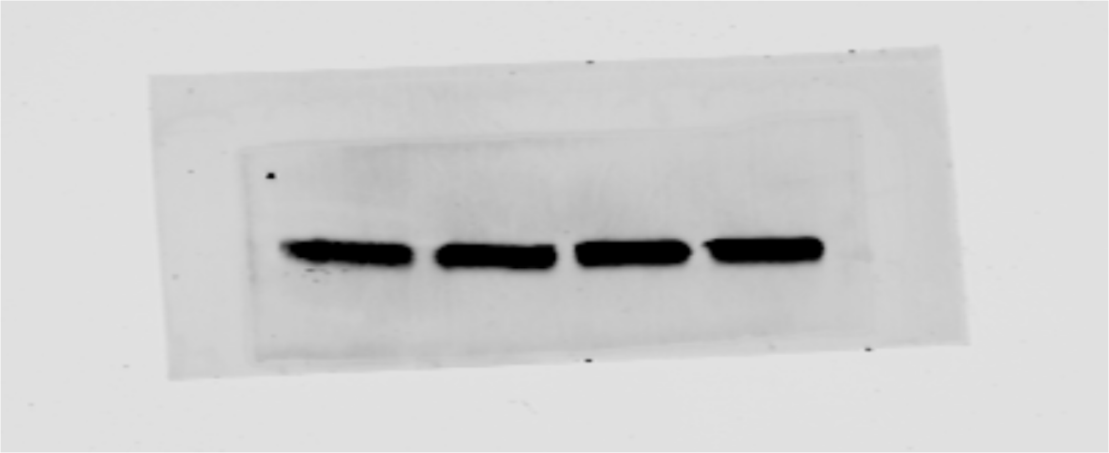

DU145 GAPDH

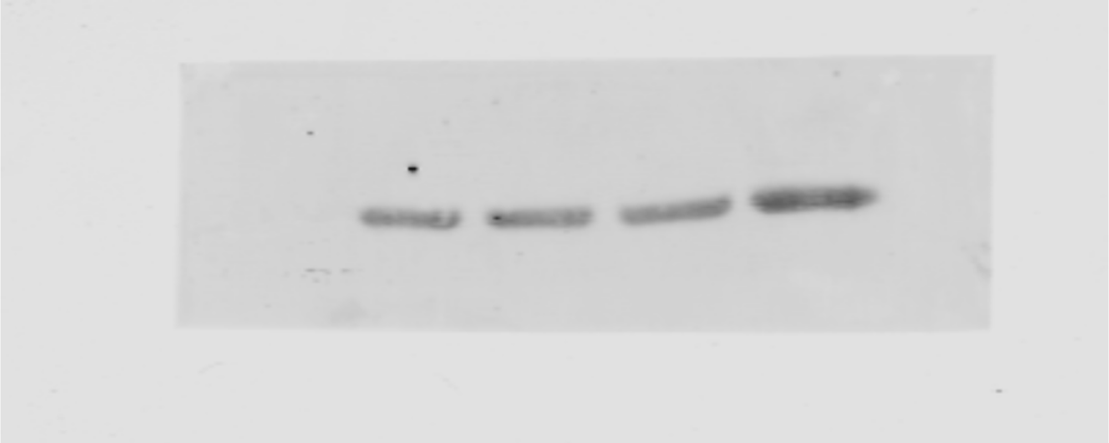

DU145 SOX15
